# Immunomodulatory amnion-derived mesenchymal stromal cells preserve muscle function in a mouse model of Duchenne muscular dystrophy

**DOI:** 10.1186/s13287-023-03337-0

**Published:** 2023-04-27

**Authors:** Yuko Nitahara-Kasahara, Soya Nakayama, Koichi Kimura, Sho Yamaguchi, Yuko Kakiuchi, Chikako Nito, Masahiro Hayashi, Tomoyuki Nakaishi, Yasuyoshi Ueda, Takashi Okada

**Affiliations:** 1 Division of Molecular and Medical Genetics, Center for Gene and Cell Therapy, The Institute of Medical Science, The University of Tokyo, 4-6-1 Shirokanedai, Minato-Ku, Tokyo, 108-8639 Japan; 2 Regenerative Medicine and Cell Therapy Laboratories, Kaneka Corporation, Kobe, Japan; 3 Department of Laboratory Medicine, The Institute of Medical Science, The University of Tokyo, Tokyo, Japan; 4 Laboratory for Clinical Research, Collaborative Research Center, Nippon Medical School, Tokyo, Japan

**Keywords:** Mesenchymal stromal cells, Amnion, Duchenne muscular dystrophy, Cell transplantation, Animal model, Anti-inflammation, Immunomodulation

## Abstract

**Background:**

Duchenne muscular dystrophy (DMD) is an incurable genetic disease characterized by degeneration and necrosis of myofibers, chronic inflammation, and progressive muscle weakness resulting in premature mortality. Immunosuppressive multipotent mesenchymal stromal cell (MSC) therapy could be an option for DMD patients. We focused on amnion-derived mesenchymal stromal cells (AMSCs), a clinically viable cell source owing to their unique characteristics, such as non-invasive isolation, mitotic stability, ethical acceptability, and minimal risk of immune reaction and cancer. We aimed to identify novel immunomodulatory effects of AMSCs on macrophage polarization and their transplantation strategies for the functional recovery of skeletal and cardiac muscles.

**Methods:**

We used flow cytometry to analyze the expression of anti-inflammatory M2 macrophage markers on peripheral blood mononuclear cells (PBMCs) co-cultured with human AMSCs (hAMSCs). hAMSCs were intravenously injected into DMD model mice (*mdx* mice) to assess the safety and efficacy of therapeutic interventions. hAMSC-treated and untreated *mdx* mice were monitored using blood tests, histological examinations, spontaneous wheel-running activities, grip strength, and echocardiography.

**Results:**

hAMSCs induced M2 macrophage polarization in PBMCs via prostaglandin E_2_ production. After repeated systemic hAMSC injections, *mdx* mice exhibited a transient downregulation of serum creatin kinase. Limited mononuclear cell infiltration and a decreased number of centrally nucleated fibers were indicative of regenerated myofibers following degeneration, suggesting an improved histological appearance of the skeletal muscle of hAMSC-treated *mdx* mice. Upregulated M2 macrophages and altered cytokine/chemokine expressions were observed in the muscles of hAMSC-treated *mdx* mice. During long-term experiments, a significant decrease in the grip strength in control *mdx* mice significantly improved in the hAMSC-treated *mdx* mice. hAMSC-treated *mdx* mice maintained running activity and enhanced daily running distance. Notably, the treated mice could run longer distances per minute, indicating high running endurance. Left ventricular function in DMD mice improved in hAMSC-treated *mdx* mice.

**Conclusions:**

Early systemic hAMSC administration in *mdx* mice ameliorated progressive phenotypes, including pathological inflammation and motor dysfunction, resulting in the long-term improvement of skeletal and cardiac muscle function. The therapeutic effects might be associated with the immunosuppressive properties of hAMSCs via M2 macrophage polarization. This treatment strategy could provide therapeutic benefits to DMD patients.

**Supplementary Information:**

The online version contains supplementary material available at 10.1186/s13287-023-03337-0.

## Background

Duchenne muscular dystrophy (DMD), the most common muscular dystrophy affecting 1 in 3500 male births, is a progressive and fatal X-linked recessive inherited skeletal and cardiac muscle disorder [1]. Dystrophin gene mutations result in the dystrophin-glycoprotein complex deficiency at the sarcolemma and progressive degeneration/regeneration cycles of the striated muscle, which is a primary degenerative myopathy with a necrotizing phase with secondary inflammation, manifesting as muscle weakness and eventual skeletal muscle atrophy [2, 3].

Steroids are widely used to improve muscle strength in patients with DMD [4, 5]. In principle, the regulation of severe inflammation in dystrophic muscle tissues could prolong the duration of therapeutic effects. However, individual differences have been observed in the beneficial effects of steroid therapy, including glucocorticoid therapy, and these compounds may have adverse effects.

Mesenchymal stromal/stem cells (MSCs) are multipotent cells derived from various tissues and express several common cell-surface antigenic markers, such as the cluster of differentiation (CD) 44, CD73, CD90, and CD105, and low levels of major histocompatibility complex (MHC) class I molecules, without expressing CD34 and CD45, hematopoietic markers, and human leukocyte antigen-DR isotype (HLA-DR), an MHC class II molecule [6]. MSCs have been extensively studied owing to their self-renewal ability and differentiation into various cell types, including osteoblasts, chondrocytes, adipocytes, and myocytes in culture [7, 8]. MSCs influence immune cell development, maturation, and function and reactive T-cell responses via cytokine and protein release [9, 10]. The multilineage potential of MSCs has been further exploited for therapeutic purposes in various diseases. Notably, the immunosuppressive properties of MSCs have been clinically applied and are commercially available for the treatment of acute graft-versus-host disease (GVHD). Therefore, MSCs may be attractive candidates for cell-based strategies for diseases characterized by acute and chronic inflammation.

Previously, we reported that bone marrow-derived multipotent mesenchymal stromal cells (BM-MSCs) could be implanted into the injured muscles of a beagle-based model of canine X-linked muscular dystrophy in Japan (CXMD_J_) [11], resulting in muscle fiber formation in the myogenic lineage [12]. However, the therapeutic effects of local treatment with BM-MSCs and the associated improvement in whole-body muscle function have not been comprehensively demonstrated. MSCs originate in the bone marrow [13], but can be isolated from numerous tissues, including adipose tissue [14], peripheral blood [15], cord blood [16], and amnion [17]. Our previous report demonstrated a safe and efficient method for isolating dental pulp-derived MSCs or dental pulp stem cells (DPSCs) [18], which can be collected non-invasively and expanded easily. Repeated systemic treatment with DPSCs enables long-term maintenance of muscle function, such as locomotor activity, in the *mdx* mouse model of DMD and CXMD_J_ [18].

Researchers are developing numerous cell therapeutic approaches to muscle recovery in patients with DMD. Nevertheless, the clinical application of these cell therapies is challenging owing to poor cell survival, limited dissemination, and inability to achieve whole-body muscle delivery. Moreover, muscle-derived stem cells can acquire dystrophin expression [19]; however, obtaining a large number of muscle-derived stem cells is challenging.

In this study, we focused on amnion-derived mesenchymal stromal cells (AMSCs), a clinically viable cell source with distinctive features, including non-invasive isolation, mitotic stability, ethical acceptability, low immunogenicity, and low risk of tumorigenesis. Human AMSCs (hAMSCs) are derived from the mesenchymal amnion layer by simple enzymatic digestion [20, 21]. In addition to pluripotency markers, hAMSCs express specific surface markers, such as CD29, CD49, CD166, stage-specific embryonic antigens (SSEA)-3/4, and common MSC markers, but do not express CD19, CD45, and CD133 [22]. hAMSCs exhibit typical MSC abilities, such as the potential for multipotent differentiation [23]. Similar to other tissue-derived MSCs, hAMSCs can exert a marked immunosuppressive effect by regulating T-cell and monocyte functions via paracrine pathways [22, 24]. Furthermore, similar to BM-MSCs, hAMSCs secrete certain growth factors, including insulin-like growth factor-1 (IGF-1), vascular endothelial growth factor (VEGF), growth differentiation factor-9 (GDF-9), and leukemia inhibitory factor (LIF), among other stem cell factors [25].

Notably, hAMSCs may be promising candidates for applications in anti-inflammatory cell therapy [26, 27], tissue engineering, and regenerative medicine, owing to their immunogenicity and stem cell properties. hAMSCs exert therapeutic effects in chemical-induced colitis [28, 29], heart failure induced by coronary artery ligation [6], GVHD [24], and cancer [30]. The advantages of using hAMSCs as a stem cell source include non-invasive procurement and a vast abundance of source material, as perinatal tissues are typically discarded after delivery.

We hypothesized that the anti-inflammatory properties of hAMSCs could ameliorate the dysfunction of dystrophic muscle and delay disease progression. Therefore, we aimed to identify novel immunomodulatory effects of hAMSCs on macrophage polarization and evaluate effective hAMSC transplantation strategies for the functional recovery of skeletal muscle using DMD models.

## Methods

### Isolation and culture of hAMSCs

Human fetal membranes were aseptically obtained from pregnant patients undergoing cesarean delivery with informed consent. Subsequently, hAMSCs were isolated and cultured according to previous studies [6, 32]. Cultured cells were harvested and frozen in liquid nitrogen until further use. hAMSCs between passages 1–5 were used for all experiments.

### Isolation of human peripheral blood mononuclear cells (hPBMCs)

We isolated hPBMCs from the blood of healthy donors with informed consent. Mononuclear cells were separated by centrifugation (20 min at 800 × g) using Lymphoprep Tube (Abbott Diagnostics Technologies, Oslo, Norway). Isolated hPBMCs were cryopreserved in liquid nitrogen until further use.

### Flow cytometry

Cell surface markers on hAMSCs or hPBMCs were analyzed using specific fluorescence-conjugated antibodies against CD11b, CD14, CD19, CD34, CD45, CD73, CD86, CD90, CD105, CD206, HLA-DR, C-X-C chemokine receptor 4 (CXCR4), and integrins α4 and β1. To prevent non-specific antibody binding, hPBMCs were pre-treated with FcR Blocking Reagent (Miltenyi Biotec, Bergisch Gladbach, Germany), according to the manufacturer’s instructions. Furthermore, 7-amino-actinomycin D (Immunostep, Salamanca, Spain) was used for dead cell exclusion. For intracellular staining, hAMSCs were fixed and permeabilized with 4% paraformaldehyde containing 0.1% polyoxyethylene (10) octylphenyl ether (Fujifilm, Osaka, Japan). Samples were analyzed using Guava easyCyte (Luminex, Austin, TX, USA). Antibody (Miltenyi Biotec) information is provided in Table 1.

**Table 1:** Antibodies used in this study

Antibody	Clone
CD11b-APC, human	M1/70
CD14-APC, human	REA599
CD19-APC, human	REA675
CD34-APC, human	AC136
CD45-APC, human	REA747
CD73-APC, human	REA804
CD86-APC, human	REA968
CD90-APC, human	REA897
CD105-APC, human	REA794
CD206-APC, human	DCN228
HLA-DR-APC, human	REA332
CD184 (CXCR4)-APC, human	REA649
CD49d (integrin α4)-APC, human	REA545
CD29 (integrin β1)-APC, human	TS2/16
REA Control (S)-APC	REA293
Mouse IgG2a Isotype Control-APC	S43.10
Rat IgG2b Isotype Control-APC	ES26-5E12.4

### Trilineage differentiation

To assess whether hAMSCs could differentiate into osteocytes, chondrocytes, and adipocytes, hAMSCs (passage 1) were cultured in respective differentiation medium, as described later in the text. To detect osteogenic differentiation, the cells were cultured in StemMACS OsteoDiff Media (Miltenyi Biotec) for 28 days and stained with Alizarin red S. For the detection of chondrogenic differentiation, cells were cultured in MesenCult-ACF chondrogenic differentiation medium (Stemcell Technologies, Vancouver, Canada) for 21 days and stained with Alcian blue. To analyze adipogenesis, cell differentiation was induced using the StemPro Adipogenesis Differentiation Kit (Thermo Fisher Scientific, Waltham, MA, USA), and the cells were stained with Oil red O. Histological features of hAMSCs were captured using a Nikon ECLIPSE TS100 microscope.

### Real-time quantitative polymerase chain reaction (qPCR)

Total RNA was extracted from hAMSCs or hPBMCs using RNeasy Mini Kit (Qiagen, CA, USA) and reverse-transcribed into cDNA using PrimeScript RT Reagent Kit (Takara Bio, Shiga, Japan), according to the manufacturer’s instructions. Real-time qPCR was performed using Taqman Fast advanced Master Mix and Taqman gene Expression assay (Thermo Fisher Scientific) on QuantStudio 7 Flex (Thermo Fisher Scientific). The following primers were used: *CD163* (Hs00174705_m1), *CXCR4* (Hs00237052_m1), glyceraldehyde-3-phosphate dehydrogenase (*GAPDH*, Hs99999905_m1), interleukin-10 (*IL10*, Hs00961622_m1), integrin subunit alpha 4 (*ITGA4*, Hs00168433_m1), *ITGB1* (Hs01127536_m1), matrix metallopeptidase 2 (*MMP2*, Hs01548727_m1), *MMP9* (Hs00957562_m1), mannose receptor C type 1 (*MRC1*, Hs00267207_m1), and transglutaminase 2 (*TGM2*, Hs01096681_m1). All primers were purchased from Thermo Fisher Scientific. Gene expression was normalized to that of *GAPDH*.

### Enzyme-linked immunosorbent assay (ELISA) of cell culture supernatants

Secreted MMP2, MMP9, and prostaglandin E_2_ (PGE_2_) concentrations in culture supernatant were detected using protein-specific ELISA kits (R&D Systems, Minneapolis, MN, USA), according to the manufacturer’s instructions. Optical density was measured using SpectraMax i3x Platform (Molecular Devices, San Jose, CA, USA).

### T cell proliferation assay

hAMSCs were treated with 10 μg/mL mitomycin C (Fujifilm) for 2 h at 37 ℃ and seeded at a density of 1 × 10^4^ cells/well in 96-well plates. After 24 h, 2 × 10^5^ hPBMCs were labeled with carboxyfluorescein succinimidyl ester (CFSE) using CFSE Cell Division Assay Kit (Cayman, Ann Arbor, MI, USA) and added to each well at a hPBMCs/hAMSCs ratio of 20:1, and the cells were co-cultured for 96 h at 37 ℃. The cells were maintained in Roswell Park Memorial Institute (RPMI) 1640 medium (Thermo Fisher Scientific) supplemented with 5% human serum (Sigma-Aldrich, St. Louis, MO, USA), 50 μM 2-mercaptoethanol (Thermo Fisher Scientific), and 1 × antibiotic–antimycotic (Thermo Fisher Scientific). To activate T cells contained within hPBMCs, 4 mg/mL phytohemagglutinin (PHA; Fujifilm) was added during co-culture. T cell proliferation after co-culture was evaluated using flow cytometry.

### Transwell co-culture

To evaluate the effect of hAMSCs on M1/M2 macrophage polarization, hAMSCs and hPBMCs were co-cultured using the Transwell system (pore size, 0.4 μm; Corning, NY, USA). hPBMCs were seeded at 2 × 10^6^ cells/well in 6-well plates, and 1 × 10^5^ hAMSCs were plated on Transwell cell culture inserts (ratio, 20:1). The cells were co-cultured in RPMI 1640 medium (Thermo Fisher Scientific) supplemented with 10% fetal bovine serum (Moregate Biotech, Bulimba, Australia) and 1 × antibiotic–antimycotic (Thermo Fisher Scientific) for 24 h. After co-culture, hPBMCs in bottom wells were harvested for flow cytometry and mRNA extraction.

To examine the secretome, in particular cytokine and chemokines, of hAMSCs under dystrophic environment, hAMSCs were co-cultured with muscle cells isolated from WT and *mdx* mice. These cells, obtained from the extensor digitorum longus (EDL) muscle using the method for isolation of individual myofibers using 5 mg/ml type I collagenase (Worthington Biochemical Corp., Lakewood, NJ, USA), were expanded in culture [33]. The muscle cells were seeded at 7.5 × 10^4^ cells/well in 24-well plates until cells were confluent (approximately 3 days), and 5.0 × 10^4^ hAMSCs were plated on Transwell cell culture inserts. These myoblasts were co-cultured in Dulbecco’s modified Eagle medium (DMEM) containing high-glucose medium (FUJIFILM Wako, Osaka, Japan), supplemented with 10% fetal bovine serum (Moregate Biotech, Bulimba, Australia), 1 × antibiotic–antimycotic (Thermo Fisher Scientific), and 1 mM pyruvate (NACALAI TESQUE, INC. Kyoto, Japan) for 5 days, and then cultured in FBS-free medium for 72 h. After co-culture, culture medium was collected for cytokine/chemokine array analysis.

### Animals

C57BL/10-background *mdx* (*mdx*), has a premature stop mutation in exon 23 of the murine *Dmd* gene, resulted to fails to translate dystrophin and mimics various aspects of the human disease [34, 35]. *Dmd*^*mdx*^ mutant and C57BL/10ScN (WT) mice were purchased from Nihon CLEA (Tokyo, Japan). All experiments were conducted in accordance with the protocols described in the experimental protocols approved by the Ethics Committee for the Treatment of Laboratory Animals at the Nippon Medical School and the Institute of Medical Science, from the University of Tokyo. All experiments were designed and reported in accordance with the Animal Research: Reporting of In Vivo Experiments (ARRIVE) guidelines. Age-matched littermate mice were used in all the experiments. Same male litter mates were housed together in individually ventilated cages with 4–6 mice per cage. Each mouse group was randomly assigned a cage location. All mice were maintained on a regular 12-h light/12-h dark diurnal lighting cycle with ad libitum access to food and water.

### Transplantation of hAMSCs into mice and sampling

Animals were randomly divided into two groups, which were control and hAMSC-treatment groups. Systemic delivery of hAMSCs (8.0 × 10^5^ cells/100 μl of PBS) into *mdx* mice via the tail vein was conducted using four injections (hAMSC-*mdx*-4, short and long term experiment, *n* = 10 and 6, each) or six injections (for long term experiment, *n* = 4) with an interval of one week between injections, beginning at 4–5 weeks of age [body weight (BW) > 10 g]. Age-matched male WT (short and long term experiment, *n* = 7, each) and *mdx* mice (short and long term experiment, *n* = 14 and 12, each) were used as controls for the experiments. The primary outcome of this study was long-term motor function as assessed by forelimb strength and running speed. The secondary endpoint was histological analysis containing inflammatory pathology. For short-term evaluations, a minimum of 10 *mdx* mice was required, as motor dysfunction was not markedly progressive in the early stages, while a smaller sample size was selected for long-term administration. The order in which each animal was tested in random, each time. At 12-, 18-week, and 1-year of age, animals were euthanized by cervical cord dislocation and tissues were extirpated for histological and molecular analysis. For analysis of heart and diaphragm, mice were sacrificed by exsanguination under 3% isoflurane (Mylan Seiyaku, Osaka, Japan) suction anesthesia and were subjected to further dissection after a satisfactory fixation by reflux fixation with 4% paraformaldehyde (FUJIFILM Wako).

### Histopathological and immunohistochemical analyses

Muscle samples were collected from hAMSC-treated mice and immediately frozen in liquid nitrogen-cooled isopentane. Subsequently, 8-μm-thick transverse cryosections were prepared from skeletal muscle, stained with hematoxylin and eosin (H&E) using standard procedures, and immunostained for macrophages. For cardiac tissue preparation, mice received perfusion fixation before tissue collection.

The tissue sections were incubated in a 3% H_2_O_2_ solution to block endogenous peroxidase for immunohistochemical analyses. The tissue sections were probed with primary antibodies anti-mouse CD206 (Bio-Rad Laboratories, CA, USA), anti-mouse F4/80 (Cedarlane Laboratories, Ontario, Canada), anti-human Ku80 (Abcam Plc., Cambridge, UK) for 1 h or overnight at 4 ℃ and then treated with 3,3′-diaminobenzidine (DAB) substrate (DAB substrate kit, Vector Laboratories Inc.) containing horseradish peroxidase (HRP) as an enzyme indicator. Subsequently, the slices were stained with DAB chromogen to determine the form of the brown-antigen reaction product. For collagen staining, cryosections were stained with Masson’s trichrome and Sirius red staining using a general protocol (Morphotecnology, Sapporo, Japan). These tissue sections were visualized using an IX71 or IX83 microscope (Olympus, Tokyo, Japan). The number of centrally nucleated fibers (CNFs) was counted visually. Whole tissue sectional images were captured from the entire slides using Nano Zoomer Digital Pathology, NPD view 2 Viewing software (HAMAMATSU Photonics K.K., Shizuoka, Japan), to avoid site-specific bias in quantitative analysis. Quantification of nucleus infiltration and collagen-stained areas was performed using CellSence software (Olympus) and image analysis software HALO (Indica Labs, Corrales, MN, USA).

### ELISA of the serum and muscle tissue lysates

Muscle tissues were disrupted using a Multi-Beads Shocker (Yasui Kikai Co., Ltd., Osaka, Japan) or Precellys 24 Tissue homogenizer (Bertin Technologies SAS, Montigny-le-Bretonneux, France) to obtain muscle tissue lysates. Creatine kinase (CK) and IL-6 expression levels were measured in the serum and tibialis anterior (TA) muscle lysates obtained from the mice using a mouse ELISA kit (R&D Systems, Minneapolis, MN, USA), according to the manufacturer’s recommendations.

### Proteome cytokine/chemokine array

Relative cytokine and chemokine expression in co-culture medium of hAMSCs and muscle cells, or muscle lysates was quantified using the Proteome Profiler™ Array (Mouse cytokine/chemokine array, Panel A; R&D Systems), as previously described [36, 37]. The blots were incubated overnight with the lysates to achieve maximum assay sensitivity. Enhanced chemiluminescence incubation was performed for 5 min using the Super Signal West Femto Chemiluminescence Kit (Thermo Scientific Pierce). The samples were imaged and analyzed using ImageQuant LAS 4000 coupled with ImageQuant TL software (GE Healthcare).

### Locomotor activity analysis

Investigators could not be blinded to the mouse strain, but the locomotor test was performed using a system to automate behavioral testing and provide unbiased data analyses. Physiological activity in the age-matched mice of each group was analyzed using a computerized wheel system (Actimo dual activity monitoring system, Shinfactory, Fukuoka, Japan) in each cage and by counting the number of wheel revolutions during 5 min intervals every 5 min using Actimo-data II software, according to our previous report [18]. Each group was randomly assigned a cage location. The order of animals to be measured within each group and the home cage location were also randomized to minimize potential confounders. The average daily running distance was calculated, and diurnal rhythm was measured for all mice over five days and nights (12 h light/dark cycles). The wheel was continuously used during observation. The data from the first day were deleted to reduce the influence of preference, as the first day was considered the habituation period. The running speed was calculated by converting m/5 min to m/min. The maximum acceleration was calculated by averaging the highest values for each of the five days.

### BW and grip strength

The BW and grip strength of age-matched mice were measured during the experiments. Forelimb grip strength was measured using a grip strength meter (MK-380 M; Muromachi Kikai, Tokyo, Japan), as previously described [38]. Five measurements were continuously recorded at 5 s intervals to rest for each mouse. The averaged force (g) was calculated for each group of mice by averaging the three highest measured values of the five consecutive values.

### Echocardiography

Mice were anesthetized by 2% isoflurane inhalation. After shaving the mouse chest, M-mode left ventricular diameters (mm) and functional shortenings (%) were evaluated using a 10 MHz linear array ultrasound transducer (i13L) with Vivid S60RN digital ultrasound system (GE Healthcare Japan, Tokyo, Japan) as described previously [36]. After analysis, mice were euthanized under anesthesia followed by tissue collection as indicated in the “Transplantation of hAMSCs into mice and sampling” section.

### Statistical analyses

For each group, data were excluded when data acquisition was difficult due to weight loss, trauma, debilitation, or death, or when there was concern about peculiar values, mechanical errors in measurement, or external environmental influences, because motor function and tissue structure could not be correctly assessed. Data are presented as the mean ± standard deviation (SD). Differences between the two groups were assessed using unpaired two-tailed t-tests. Multiple comparisons between three or more groups were performed using analysis of variance (ANOVA, *n* = 3–6, Tukey’s post hoc test). Statistical significance was defined as ^*^P < 0.05, ^**^P < 0.01, ^***^P < 0.001, and ^****^P < 0.0001 and was calculated using GraphPad Prism 8 or 9 (GraphPad, La Jolla, CA, USA).

## Results

### Characterization of hAMSCs

**Fig. 1:**
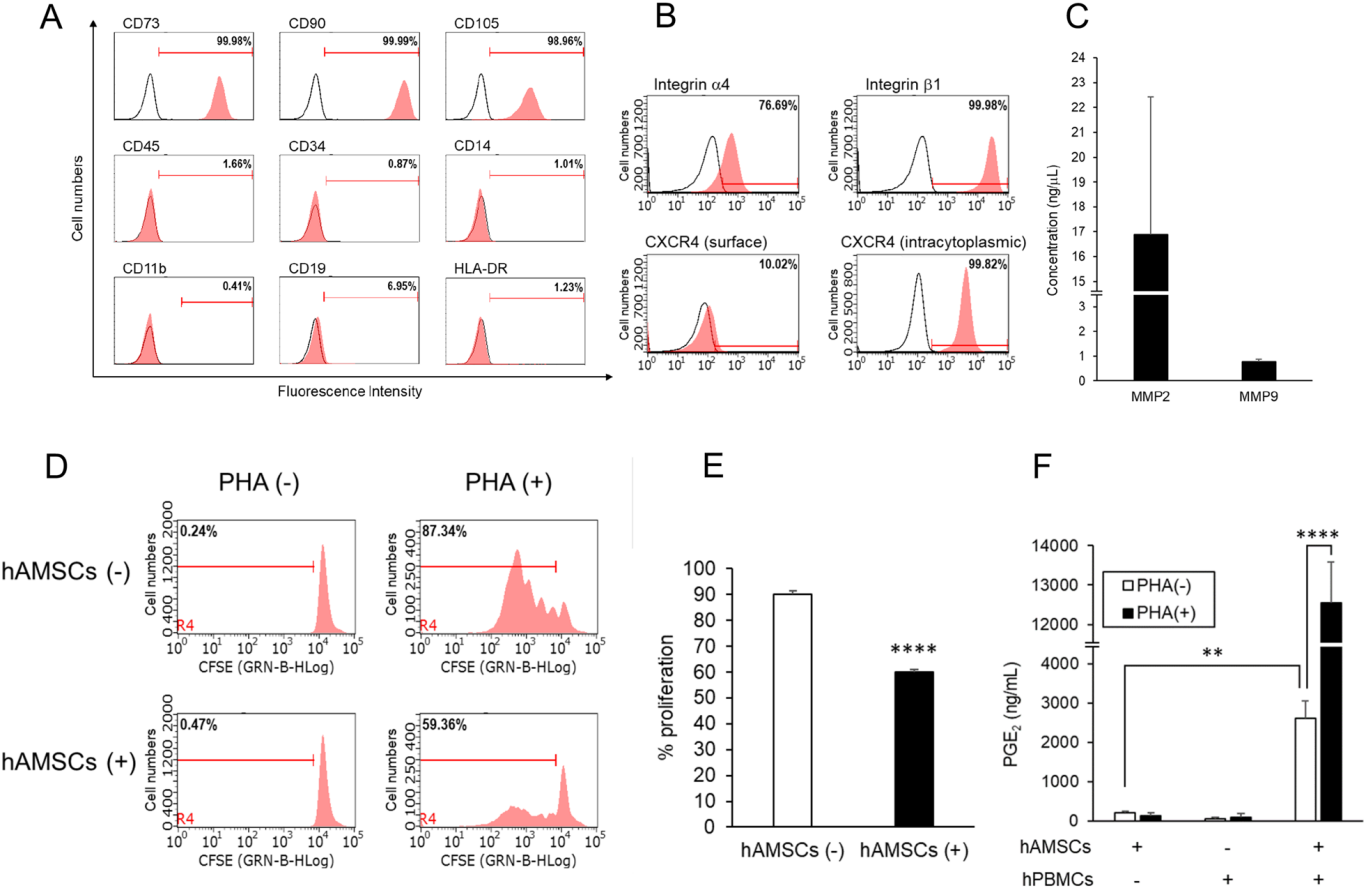
Immunophenotypic, migratory, and immunosuppressive characteristics of hAMSCs. **A** Surface marker expression on hAMSCs was detected using flow cytometric analysis. The cells were positive for CD73, CD90, and CD105, common MSC markers, and negative for CD45, CD34, CD14, CD11b, CD19, and HLA-DR. **B** Integrin α4, integrin β1, and CXCR4 expression were detected using flow cytometric analysis. White curves represent isotype controls. **C** MMP2 and MMP9 secretion was detected using ELISA. Data are presented as the mean ± SD of three independent experiments. **D**, **E** hAMSCs were co-cultured with PHA-activated or non-activated hPBMCs at a ratio of 1:20. hPBMC proliferation was determined after four days using flow cytometry. A representative experiment (**D**), the mean ± SD of three independent experiments (**E**). **F** Culture supernatants were collected 24 h after seeding and assayed for PGE_2_ production. All data are presented as the mean ± SD of three independent experiments and statistical differences (^**^P < 0.01, ^****^P < 0.0001) are shown

We obtained hAMSCs from healthy amnion as previously described [6]. Cultured hAMSCs were positive for CD73, CD90, and CD105, typical MSC markers [31], but negative for hematopoietic markers (Fig. 1A). The cells exhibited fibroblastic morphology (Additional file 1: Figure S1A) and could differentiate into osteocytes and chondrocytes (Additional file 1: Figure S1B–C). Furthermore, hAMSCs did not have adipogenic differentiation potential (Additional file 1: Figure S1D), consistent with previous studies [23]. These results indicated that hAMSCs had common multipotent MSC characteristics.

### hAMSCs exhibited migratory and immunomodulatory properties

Cell migration and immunomodulation are well-known therapeutic properties of MSCs [39, 40]. Therefore, we investigated these properties in hAMSCs. To assess the migratory potential of hAMSCs, we examined the expression levels of CXCR4, integrins α4 and β1, MMP2, and MMP9, cell migration-related factors [41, 42]. Notably, hAMSCs expressed these cell migration-related factors at the mRNA level compared with normal human dermal fibroblasts (NHDF) and human bone-marrow MSC (hBM-MSC) (Additional file 1: Figure S2A), wherein ITGA4, ITGB1, and MMP2 were commonly expressed in both MSCs to a similar degree, and CXCR4 was expressed slightly higher from hAMSCs than BM-MSCs. Overall, hAMSCs showed a similar pattern of expression of migration-related factors as NHDFs. CXCR4, and integrins α4 and β1 expression was detected using flow cytometric analysis (Fig. 1B). Furthermore, hAMSCs expressed low CXCR4 levels on the surface but high CXCR4 levels in the cytoplasm. MMP2 and MMP9 secretion was detected using ELISA (Fig. 1C). The amount of secreted MMP9 was much lower than that of MMP2, as described in previous studies [43]. These data demonstrated the migratory potential of hAMSCs.

The immunomodulatory ability of hAMSCs was investigated using a T cell proliferation assay. PHA-activated T cell proliferation was significantly inhibited when hPBMCs were co-cultured with hAMSCs (hAMSCs (-), 90.1 ± 1.4% of hPBMCs proliferated; hAMSCs ( +), 59.9 ± 1.2% of hPBMCs proliferated, *P* < 0.0001) (Fig. 1D–E). Additionally, hAMSCs co-cultured with hPBMCs, particularly those co-cultured with PHA-activated hPBMCs, secreted PGE_2_, one of the main immune modulators produced by MSCs [39, 44] (hPBMCs (-), 214.2 ± 48.9 ng/μL; hPBMCs ( +), 2619.1 ± 434.8 ng/μL, *P* = 0.009; PHA-activated hPBMCs ( +), 12,548.8 ± 1030.3 ng/μL, *P* < 0.0001) (Fig. 1F).

### hAMSCs increased M2 macrophage marker expression levels via PGE_2_ production in vitro

**Fig. 2:**
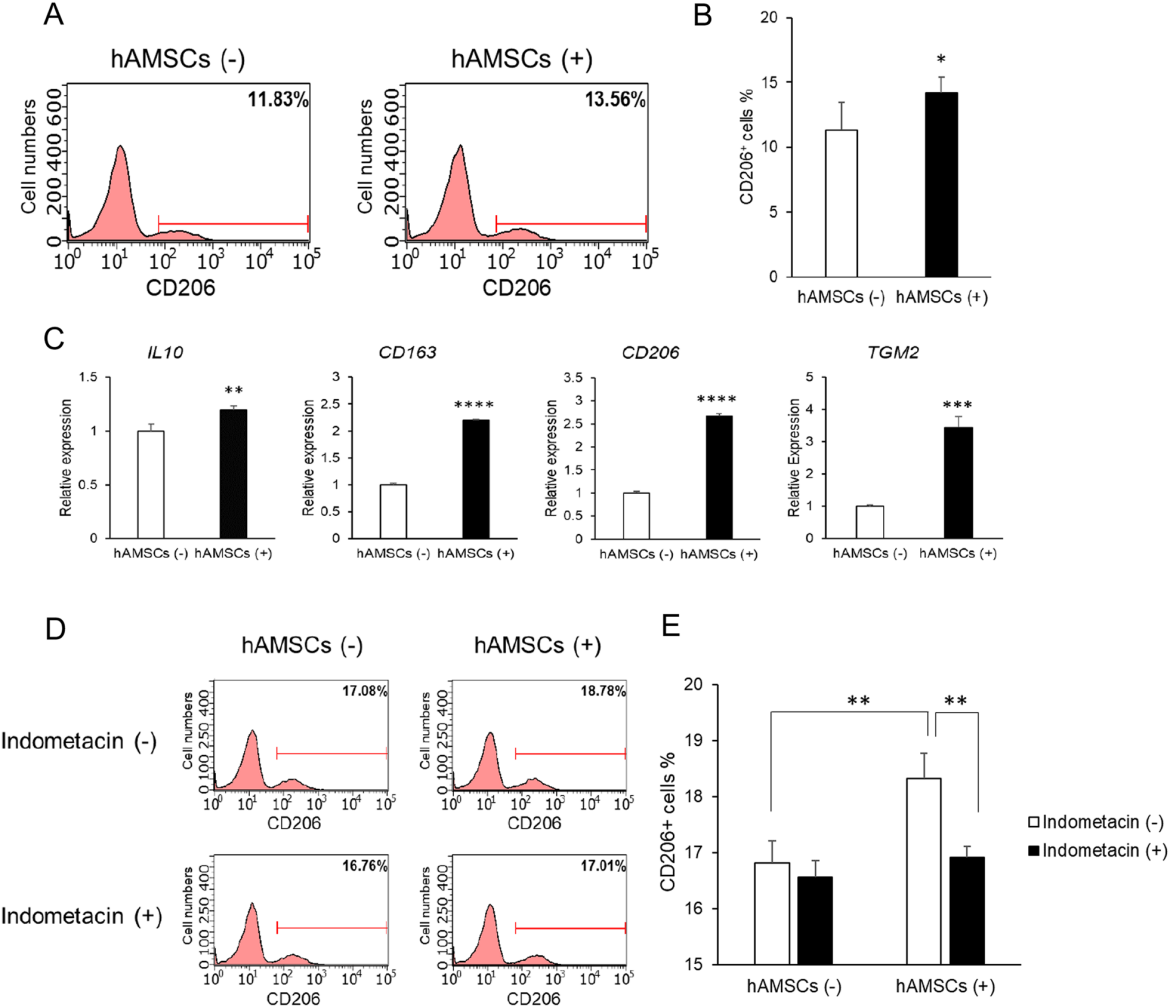
hAMSCs induce M2 macrophage polarization via PGE_2_ production. **A**, **B** hAMSCs were co-cultured with hPBMCs at a ratio of 1:20. CD206 expression in hPBMCs was determined after 24 h using flow cytometry. A representative experiment (**A**) and the mean ± SD of three independent experiments (**B**) are shown. **C** Relative mRNA levels of M2 macrophage markers in hPBMCs with or without hAMSCs co-culture. Relative expression was obtained after normalizing the mRNA levels to those in hPBMCs without MSC co-culture. Data are presented as the mean ± SD of three independent experiments. **D**, **E** hAMSCs were co-cultured with PHA-activated hPBMCs at a ratio of 1:20 in the presence or absence of indomethacin, a PGE_2_ production inhibitor. CD206 expression in hPBMCs was determined after 24 h using flow cytometry. A representative experiment (**D**), the mean ± SD of three independent experiments (**E**). Statistical differences are shown as ^*^P < 0.05, ^**^P < 0.01, ^***^P < 0.001, and ^ ****^P < 0.0001

The M1/M2 paradigm comprises an M1 component with pro-inflammatory cytokine and chemokine secretion and an M2 component with immunoregulatory, wound healing, and fibrotic properties [45]. Moreover, MSCs derived from several tissues induce anti-inflammatory M2 macrophages [46–48]. Thus, we investigated whether hAMSCs could induce M2 macrophage polarization. hPBMCs were co-cultured with hAMSCs using the Transwell system, and the expression rate of CD206, a specific M2 macrophage marker, in hPBMCs was evaluated (hAMSCs (-), 11.3 ± 2.2%; hAMSCs ( +), 14.2 ± 1.3%, *P* = 0.035) (Fig. 2A–B). CD206 expression rate was slightly increased in the co-culture system. However, the expression rate of inflammatory M1 macrophages, which are CD86-positive, was not increased (hAMSCs (–), 17.6 ± 0.5%; hAMSCs (+), 16.6 ± 0.7%, *P* = 0.069) (Additional file 1: Figure S2). Furthermore, hPBMCs-hAMSCs co-culture promoted the mRNA expression of M2 macrophage markers, including *IL10* (relative expression compared with hAMSC(-): 1.2 ± 0.04, *P* = 0.006), *CD163* (2.2 ± 0.01, *P* < 0.0001), *MRC1* (2.7 ± 0.06, *P* < 0.0001), and *TGM2* (3.4 ± 0.3, *P* = 0.0001) (Fig. 2C). These results demonstrated that hAMSCs preferentially induced M2 macrophages in hPBMCs.

Subsequently, we explored a mediator of M2 macrophage induction by hAMSCs. PGE_2_ is one of the major anti-inflammatory mediators secreted by MSCs. We revealed that hAMSCs secreted PGE_2_ in the hAMSCs-hPBMCs co-culture system (Fig. 1F). To examine the effect of PGE_2_ on M2 macrophage induction, we used indomethacin, a PGE_2_ production inhibitor. Indomethacin attenuated the increase in CD206 expression rate in hPBMCs caused by hAMSCs (hAMSCs (–), 16.8 ± 0.4%; hAMSCs (+), 18.3 ± 0.5%, *P* = 0.009; hAMSCs ( +) and indomethacin ( +), 16.9 ± 0.2%, *P* = 0.007) (Fig. 2D–E). These data suggested that hAMSCs induced M2 macrophage polarization in hPBMCs via PGE_2_ production.

### Effects of systemic hAMSC administration on DMD mice

**Fig. 3:**
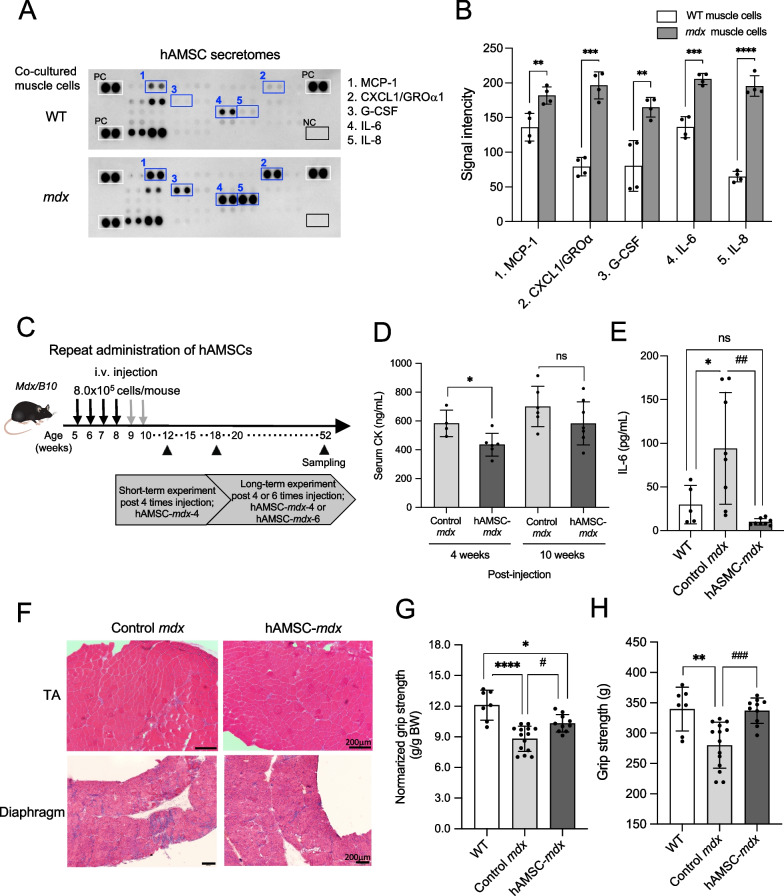
Establishment and evaluation of systemic hAMSC administration using DMD mice. **A** Cytokine and chemokines expression in 3-day co-culture media of hAMSCs with muscle cells from WT (B10) or *mdx* mice was analyzed using the Proteome Profiler^TM^ Array. Changes in the expression levels circled with blue squares 1–5 are monocyte chemotactic protein-1 (MCP-1), chemokine (C-X-C motif) ligand 1(CXCL1)/growth-regulated protein α (GROα), granulocyte colony stimulating factor (G-CSF), IL-6, and IL-8, compared with WT cells. PC; positive control signals, NC; negative control. **B** Data quantifying signal intensity for the dots circled with blue squares in the array image (**A**). **C** Schematic representation of the repeated hAMSC treatment of *mdx* mice. For the short-term evaluation, mice received four treatments (hAMSC-*mdx*-4), whereas, for the long-term evaluation, we examined two groups that had received a total of four, or six treatments (hAMSC-*mdx*-4 or hAMSC-*mdx*-6). **D** Serum creatine kinase (CK) levels in each group of mice; 12- and 18-week-old mice of control *mdx* (*n* = 4, and 6), and four-time hAMSC-*mdx* (*n* = 6, and 7), using ELISA. **E** Quantification of IL-6 levels in the TA muscle lysate (300 μg protein) of wild type (WT, *n* = 3), control *mdx* (*n* = 5), and four-time hAMSC-*mdx* (*n* = 4) mice using ELISA. **F** Hematoxylin and eosin (**H**, **E**) staining of the TA and diaphragm muscle (original magnification, × 100 and × 40, each) of control *mdx* and four-time hAMSC-*mdx*. Scale bars, 200 μm. **G** Grip strength (g) and **H** normalized grip strength (g/g body weight, BW) of 12-week-old WT (*n* = 7), control *mdx* (*n* = 14)*,* and four-time hAMSC-treated *mdx* (*n* = 10) mice. All data are presented as the mean ± SD, statistical differences are expressed relative to WT (^*^P < 0.05, ^**^P < 0.01, ^***^P < 0.0005 and ^****^P < 0.0001) and control *mdx* (^#^*P* < 0.05, ^##^*P* < 0.01, and ^###^*P* < 0.0005) mice, t-test (**D**), and one-way ANOVA (Tukey’s post hoc test; **E**, **G**, **H**)

To investigated the efficiency of hAMSCs administration under dystrophic environment, we analyzed extracellular secretion of cytokine and chemokine in the co-cultured medium of hAMSCs with myocytes (Fig. 3A, B). We confirmed that monocyte chemotactic protein-1 (MCP-1, WT *vs. mdx*;* P* = 0.008), chemokine (C-X-C motif) ligand 1(CXCL1)/ growth-regulated protein α (GROα,* P* < 0.0001), granulocyte colony stimulating factor (G-CSF,* P* = 0.005), IL-6 (*P* = 0.0002), and IL-8 (*P* < 0.0001) were significantly up-regulated in the co-cultured medium of hAMSCs with myocytes derived from *mdx* mice, compared with the levels observed in the medium from co-cultured WT muscle cells. These results suggested that the secretion from hAMSCs was enhanced in the dystrophic environment of myoblasts.

To examine the effect of hAMSCs on DMD model mouse, we repeatedly administered hAMSCs into *mdx* mice via the tail vein during the acute phase of the disease (Fig. 3C). For the short-term evaluation, mice received four treatments (hAMSC-*mdx*-4), whereas, for the long-term evaluation, we examined two groups that had received a total of four, or six treatments (hAMSC-*mdx*-4 or hAMSC-*mdx*-6). We first confirmed that the BW of hAMSC-treated *mdx* mice did not significantly vary during the experiments (Additional file 1: Figure S3), indicating that repeated hAMSC-treatment did not adversely affect the *mdx* mice.

The serum levels of CK, a DMD marker, were transiently reduced in four-time hAMSC-*mdx* mice until four weeks after injection (435.2 ± 79.3 ng/mL) compared to those in age-matched *mdx* mice (582.7 ± 92.1 ng/mL, *P* = 0.038), but this effect was transient (10 weeks post hAMSC treatment, 582.0 ± 149.2 ng/mL and without treatment, 699.9 ± 140.6 ng/mL, *P* = 0.228; Fig. 3D). Furthermore, the pro-inflammatory cytokine IL-6 was downregulated in the skeletal muscle of hAMSC-treated *mdx* mice (10.0 ± 3.8 pg/mL) compared to that of untreated mice (94.2 ± 64.1 pg/mL, *P* = 0.002; Fig. 3E). We then performed histological analysis to assess the effects of four-time hAMSC treatment on tissue structure. The cross-section of the TA muscle of *mdx* mice revealed spread muscle interstitium, reflecting muscle fiber size variation and cells dispersed in the muscle interstitium (Fig. 3F). The histopathological examination of the TA and diaphragm muscles of hAMSC-treated *mdx* mice revealed little mononuclear cell infiltration and muscle interstitium, indicating a milder dystrophic phenotype. Furthermore, we evaluated the muscle function of *mdx* mice by measuring the grip strength (280.0 ± 37.8 g; normalized grip strength, 8.8 ± 1.2 g/g BW), which was significantly restored after repeated hAMSC administration (336.8 ± 21.0 g, *P* = 0.0008; 10.3 ± 0.9 g/g BW, *P* = 0.012; Fig. 3G, H). Therefore, we observed a short-term improvement of dystrophic muscle after repeated systemic treatment with hAMSCs.

### Immunomodulatory effects of repeated hAMSC treatment on dystrophic muscle

**Fig. 4:**
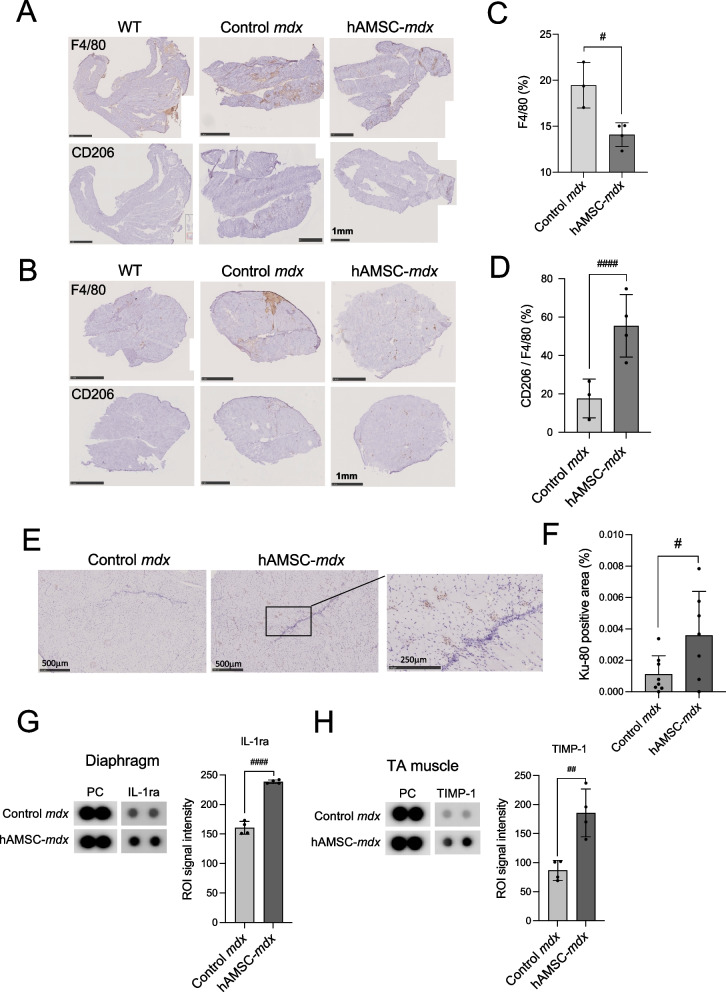
Regulatory effects of repeated hAMSC treatment on DMD-associated inflammation in skeletal muscle. **A**, **B** Horseradish peroxidase (HRP)-diaminobenzidine (DAB) immunostaining for F4/80 and CD206 in the diaphragm (**A**) and tibialis anterior (TA) muscle (**B**) of 18-week-old WT, control *mdx,* and four-time hAMSC-treated *mdx* mice (hAMSC-treated *mdx*-4). Original magnification, × 50. Scale bars, 1 mm. **C** Quantification of F4/80-positive area in the cross-section (% of total area) of the diaphragm of control *mdx* (*n* = 3) and four-time hAMSC-treated *mdx* (*n* = 4) mice. **D** Quantification of the ratio of CD206-positive area in the cross-section (% of F4/80 staining area) of the TA muscle of control *mdx* (*n* = 3)*,* and four-time hAMSC-treated *mdx* (*n* = 4) mice. **E** Horseradish peroxidase (HRP)-diaminobenzidine (DAB) immunostaining for human Ku80 in the tibialis anterior (TA) muscle of 12-week-old WT, control *mdx,* and four-time hAMSC-treated *mdx* mice (hAMSC-treated *mdx*-4). Original magnification, × 80. Scale bars, 500 μm. High-magnification images represent the boxed regions in the hAMSC-*mdx* (left panel). Original magnification, × 200. Scale bar, 250 μm. **F** Quantification of the ratio of Ku80-positive area in the cross-section area of the control *mdx* (*n* = 8) TA muscle*,* and four-time hAMSC-treated *mdx* (*n* = 7) mice. **G**, **H** Cytokine and chemokine expression in the diaphragm (**G**) and TA muscle (**H**) of 12-week-old WT, control *mdx,* and four-time hAMSC-treated *mdx* mice were analyzed using the Proteome Profiler™ Array. The signals cropped from original images in Additional file 1: Figure S7 show changes in the expression levels of interleukin-1 receptor antagonist (IL-1ra), and tissue inhibitor of metalloproteinases-1 (TIMP-1) relative to those of positive control (PC) signals. Signal intensity in the regions of interest (ROIs) was quantified using array images (left panels), and representative data (graph) are presented. All data are presented as the mean ± SD, statistical differences are expressed relative to control *mdx* mice (^#^*P* < 0.05, ^##^*P* < 0.01 and ^####^*P* < 0.0001, t-test)

We assessed macrophage accumulation and polarization to examine the short-term immunomodulatory effect of repeated hAMSC treatment on dystrophic muscle. Histopathological analysis of muscle tissue revealed a noticeable accumulation of F4/80-positive macrophages, which was reduced in the diaphragm muscle of four-time hAMSC-treated *mdx* mice (Fig. 4A). Small amounts of cells positive for CD206, an M2 macrophage marker, were present at the site of F4/80-positive cell accumulation in the TA muscle tissue of *mdx* mice (Fig. 4B). Quantitative analysis revealed that hAMSC-treated mice displayed a low accumulation of F4/80-positive cells (control *mdx*, 19.5 ± 2.5%; hAMSC-*mdx*-4, 14.0 ± 1.3%, *P* = 0.013) in the diaphragm (Fig. 4C) and a higher proportion of CD206^+^ per F4/80^+^ cell ratio (control *mdx*, 17.6 ± 10.1%; hAMSC-*mdx*-4, 55.5 ± 16.3%, *P* < 0.0001) in the TA muscle than untreated mice did (Fig. 4D), suggesting that anti-inflammatory M2 macrophages were slightly predominant in hAMSC-treated muscle.

When we examined the retention of hAMSCs in the four-time hAMSC-treated *mdx* mice, hAMSCs were observed in the TA muscle 4-week after treatments, as revealed by Ku80 antibodies specific for human cell nuclei (Ku80^+^ area in hAMSC-*mdx*-4 muscle sections, 0.0036 ± 0.0028%; vs. control *mdx*,* P* = 0.036; Fig. 4E, F), but not detected in the diaphragm (*P* = 0.44). The Ku80^+^ cells were diminished 10-week after hAMSC treatment (*P* = 0.28).

To determine immunomodulatory myokine expression induced by the four-time hAMSC treatment, cytokine and chemokine arrays were performed using the TA and diaphragm muscles of each mouse group (Fig. 4E, F). Cropped array images depict IL-1 receptor antagonist (IL-1ra) levels in the diaphragm of *mdx* mice, which were upregulated in hAMSC-treated mice (Fig. 4E). In addition, the TA muscle of hAMSC-treated mice had increased tissue inhibitor of metalloproteinases-1 (TIMP-1) expression levels (Fig. 4F). Full-length blots are presented in Additional file 1: Figure S4. These results demonstrated the immunosuppressive effect of hAMSC treatment on dystrophic muscle.

Moreover, dystrophin expression in the TA and diaphragm muscles was not detected using reverse transcription PCR, suggesting that hAMSCs did not recover dystrophin expression via their myogenic differentiation (Additional file 1: Figure S5).

### hAMSC treatment resulted in long-term improvements in locomotor activity and histopathological features of *mdx* mice

**Fig. 5:**
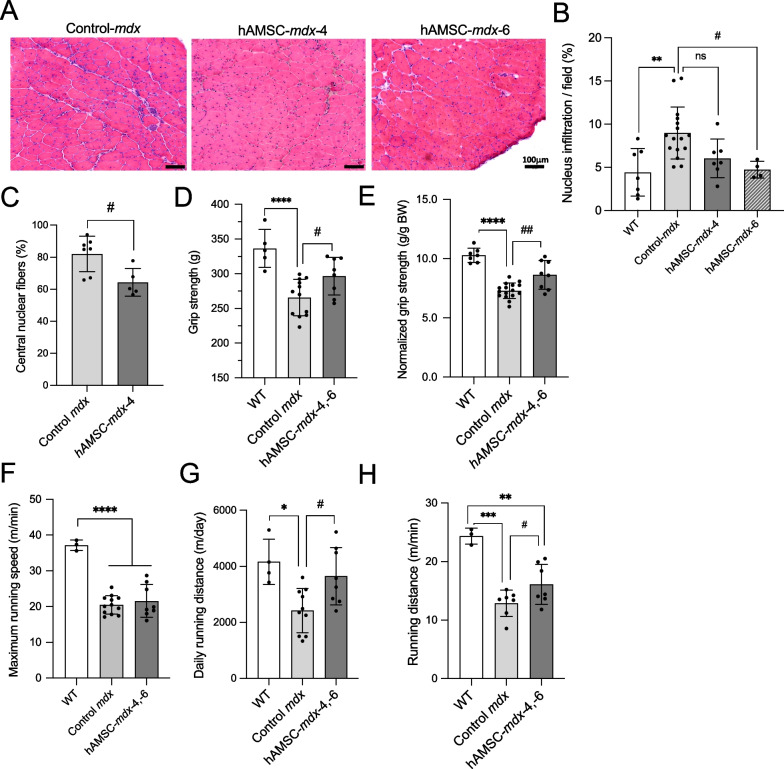
Successful long-term maintenance of muscle function in hAMSC-treated DMD mice. **A** Hematoxylin and eosin (H&E) staining of the tibialis anterior (TA) muscle of 1-year-old control *mdx* and hAMSC-treated *mdx* (four or six time treatments; hAMSC-*mdx*-4 or hAMSC-*mdx*-6) mice. Original magnification, × 100. Scale bars, 200 μm. **B** Quantification of mononuclear cell infiltration in the cross-section (% of total area) of the TA muscle of 1-year-old WT (*n* = 7), control *mdx* (*n* = 14)*,* and hAMSC-treated *mdx* (hAMSC-*mdx*-4 or -6; *n* = 4, each) mice. **C** Quantification of CNFs (% of total area) of the TA muscle of 1-year-old control *mdx* (*n* = 7) and four-time hAMSC-treated *mdx* (hAMSC-*mdx*-4, *n* = 5) mice. **D** Grip strength (gram, g) and **E** normalized grip strength (g/g body weight, BW) of 1-year-old WT (*n* = 7), control *mdx* (*n* = 12), and 4 and 6 times hAMSC-treated *mdx* mice (hAMSC-*mdx*-4, -6, *n* = 8). **F**, **G**, **H** Voluntary running activity in 1-year-old WT, control *mdx* (*n* = 10), and hAMSC-treated *mdx* mice reached the (**F**) maximum running speed (m/min; WT, *n* = 3; control *mdx*, *n* = 12; hAMSC-*mdx*-4, -6, *n* = 8), **G** daily running distance (m/day; WT, *n* = 4; control *mdx*, *n* = 10; hAMSC-*mdx*-4, -6, *n* = 8) and **H** average running distance (m/min; WT, *n* = 3; control *mdx*, *n* = 5; hAMSC-*mdx*-4, -6, *n* = 5). All data are presented as the mean ± SD; statistical differences are expressed relative to WT (^*^P < 0.05, ^**^P < 0.01, ^***^P < 0.001, and ^****^P < 0.0001) and control *mdx* (^#^*P* < 0.05, and ^##^*P* < 0.01) mice; ns, not significant; one-way ANOVA (Tukey’s post hoc test)

We investigated the long-term effects of four- or six-time hAMSC treatment on 1-year-old mice. Numerous CNFs were observed in dystrophic muscles, which indicated regenerated myofibers following degeneration, spread muscle interstitium, and cells dispersed in the muscle interstitium, as demonstrated in H&E images (Fig. 5A–C, Additional file 1: Figure S7A, B). Quantitative analysis revealed that the reduction in nuclear area was observed only in the six-time hAMSC-treated TA muscle compared to that in untreated *mdx* mice (control *mdx*, 9.0 ± 3.0%; hAMSC-*mdx*-4, 7.3 ± 1.9%, hAMSC-*mdx*-6, 4.7 ± 1.0%; control *mdx* vs*.* hAMSC-*mdx*-6, *P* = 0.038; Fig. 5B), but not significant difference in the diaphragm (Additional file 1: Figure S7A, B). Furthermore, dystrophic TA muscle exhibited a reduced number of CNFs after 4 and 6 times of hAMSC administration (control *mdx*, 82.0 ± 11.1%; hAMSC-*mdx-*4, -6, 64.3 ± 8.6%, *P* = 0.014), suggesting immunosuppressive effects of hAMSCs (Fig. 5C). However, we did not detect significant differences in the fibrotic area in the TA muscle (Additional file 1: Figure S6A, B), diaphragm (Additional file 1: Figure S7A, C), and heart (Additional file 1: Figure S8A, C) using histopathological analysis, suggesting that fibrosis was not affected by repeated hAMSC treatment.

Strikingly, the grip strength of four- and six-time hAMSC-treated *mdx* mice (hAMSC-*mdx*-4, -6: grip strength, 296.5 ± 27.2 g; grip strength normalized to BW, 8.6 ± 1.2 g/g BW) was maintained compared to that of *mdx* mice (265.7 ± 26.3 g, *P* = 0.049; 7.3 ± 0.6 g/g BW, *P* = 0.002) but did not reach that of WT mice (336.4 ± 27.4 g; 10.3 ± 0.6 g/g BW; Fig. 5D, E). We observed no significant difference in BW between the 1-year-old mice of different groups (Additional file 1: Figure S3B). The number of F4/80-positive macrophages was unchanged in 1-year-old control *mdx* and four- and six-time hAMSC-treated *mdx* mice (hAMSC-*mdx*-4, -6), whereas a slightly larger number of CD206-positive cells was detected in the TA muscle (Additional file 1: Figure S6C).

Furthermore, we examined progressive resistance against wheel running dysfunction in the hAMSC-treated DMD mice (Fig. 5F–H). The maximum running speed of four- and six-time hAMSC-treated (21.6 ± 4.6 m/min) and untreated (20.5 ± 2.6 m/min, *P* = 0.77) *mdx* mice was lower than that of WT mice (Fig. 5F); however, the daily running distance (3649 ± 1025 m/day) of four- and six-time hAMSC-treated *mdx* mice was greater than that of *mdx* mice (2420 ± 787.6 m/day, *P* = 0.028; Fig. 5G). Importantly, four- and six-time hAMSC-treated *mdx* mice (hAMSC-*mdx*-4, -6) had a greater running distances per minute (18.3 ± 3.3 m/min) than control *mdx* mice did (14.7 ± 2.6 m/min, *P* = 0.047) in the wheel (Fig. 5H). The histogram in Additional file 1: Figure S9A depicts the increased running distance of four- and six-time hAMSC-treated *mdx* mice (hAMSC-*mdx*-4, -6) and the highest rate of running distance (WT, 40.4 m/min; control *mdx*, 22.1 m/min; hAMSC-treated *mdx*-4, -6, 30.9 m/min). Moreover, all mouse groups had a similar horizontal activity, which was measured for 24 h using a beam sensor in the home cage (Additional file 1: Figure S9B). However, circadian locomotor rhythm indicated that horizontal locomotor activity was reduced at night, and the wheel running activity of four- and six-time hAMSC-treated *mdx* mice (hAMSC-*mdx*-4, -6) was increased compared to that of control *mdx* mice (Additional file 1: Figure S9C, and S9D). Collectively, these findings indicated that the four- and six-time hAMSC administration delayed the progression of muscle dysfunction in *mdx* mice, as the locomotor activity of hAMSC-treated mice was maintained until the mice were 12 months old.

### Cardiac improvement in hAMSC-treated mice

**Fig. 6:**
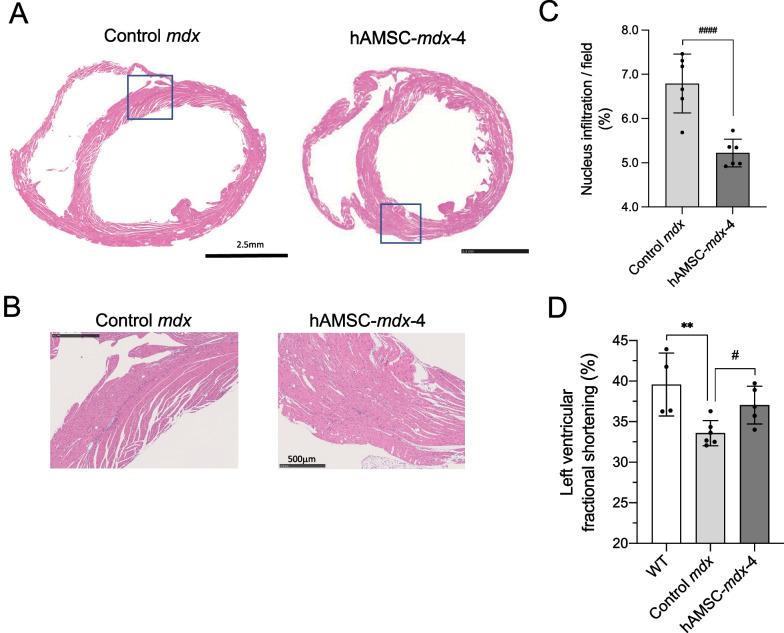
Improved cardiac function in hAMSC-treated DMD mice. **A** Hematoxylin and eosin (H&E) staining of short-axis ventricular cross-sections of 1-year-old control *mdx* and four-time hAMSC-treated *mdx* (hAMSC-*mdx*-4) mice. Original magnification, × 27. Scale bars, 2.5 mm. **B** High-magnification images represent the boxed regions in the upper A panels. Original magnification, × 200. Scale bars, 500 μm. **C** Quantification of mononuclear cell infiltration in the short-axis ventricular cross-section (% of total area) of 1-year-old control *mdx* (*n* = 6) and hAMSC-treated *mdx* (hAMSC-*mdx*-4, *n* = 6) mice. **D** Left ventricular fractional shortening (%) was calculated by M-mode echocardiography (WT, *n* = 4; control *mdx*, *n* = 6; hAMSC-*mdx*-4, *n* = 5). All data are presented as the mean ± SD, statistical differences are expressed relative to WT (^**^P < 0.01) and control *mdx* (^#^*P* < 0.05 and ^####^*P* < 0.0001) mice (t-test)

We investigated whether repeated systemic administration of hAMSC could improve progressive cardiomyopathy (Fig. 6). Histological analysis of the heart tissues revealed that morphological characteristics did not significantly differ between control *mdx* and hAMSC-treated *mdx* mice (Fig. 6A). No significant differences were observed in altered M1/M2 macrophage polarizations (Additional file 1: Figure S8B, D, E).

However, a reduced mononuclear cell infiltration was observed in the four-time hAMSC-treated *mdx* mice (Fig. 6B, C). Echocardiography revealed a higher left ventricular fractional shortening in hAMSC-treated *mdx*-4 mice (mean ± SD, 37.0 ± 2.3%) than those in untreated *mdx* mice (33.6 ± 1.6%, *P* = 0.017), which was comparable to those in WT mice (39.6 ± 3.9%, Fig. 6D and Table 2). Collectively, these results demonstrated that hAMSC treatment attenuated inflammation and ameliorated the progression of cardiomyopathy.

**Table 2:** Echocardiography results

Variable	WT (*n* = 4)	Control *mdx* (* n* = 6)	hAMSC*-mdx-*4 (*n* = 8)	*P*-value	
				WT versus control *mdx*	Control *mdx* versus hAMSC-*mdx-*4
IVSd (mm)	1.0 ± 0.1	1.1 ± 0.1	1.1 ± 0.2	NS	NS
LVPWd (mm)	1.2 ± 0.2	1.2 ± 0.1	1.2 ± 0.4	NS	NS
LVDd (mm)	3.5 ± 0.1	3.7 ± 0.2	3.6 ± 0.2	NS	NS
LVFS (%)	39.6 ± 3.9	33.6 ± 1.9	37.0 ± 2.3	0.008	0.017

IVSd, interventricular septal thickness at end-diastole; LVPWd, left ventricular posterior wall thickness at end-diastole; LVDd, left ventricular diameter at end-diastolic; LVFS, left ventricular fractional shortening at end-diastole were calculated by M-mode echocardiography of WT (*n* = 4), control *mdx* (*n* = 6), and four-time hAMSC treated-*mdx* (hAMSC-*mdx*-4, *n* = 5). Data are presented as the mean ± SD; statistical differences are expressed relative to wild-type (WT) or control *mdx* mice; NS, not significant; t-test

## Discussion

In this study, we revealed that hAMSCs co-cultured with macrophages induced M2 macrophage polarization by secreting anti-inflammatory cytokines. We hypothesized that immunosuppressive hAMSCs might be attractive candidates for cell-based therapies for DMD, characterized by chronic inflammation and progressive muscle dysfunction. Therefore, we designed the strategies for safe and effective hAMSC treatment using a DMD mouse model at an early disease stage to assess the functional recovery of skeletal muscle.

Recent studies have reported that hAMSCs exert immunosuppressive effects in co-culture with T-cells [49], in which indoleamine 2,3-dioxygenase (IDO) secreted by hAMSCs induces immunosuppressive effects in the same manner as BM-MSCs [49]. Additionally, a previous report demonstrated the anti-proliferative effects of the hAMSC-conditioned medium on lymphocytes and monocytes [50]. Furthermore, macrophage phagocytosis of BM-MSCs is greatly enhanced [51], and IL-10 production is increased under LPS stimulation [52]. MSC-mediated macrophage polarization has been demonstrated in various inflammatory diseases and is regulated by several mediators secreted by MSCs, such as IDO [53, 54], tumor necrosis factor-alpha-induced protein 6 (TSG-6) [55], IL-6 [56] and PGE_2_ [57, 58]. Production of PGE_2_ in the MSC secretome is a major paracrine mediator instructing M1 to M2 functional and bioenergetics shifts in co-cultures [58]. However, the correlation between hAMSCs and macrophage activity has been less explored. As macrophages polarize to M1 or M2 subsets with different effector functions in response to their microenvironment [59], we analyzed M2 macrophage polarization to assess whether MSC treatment altered macrophage phenotypes. We first demonstrated the influence of hAMSCs on macrophage polarization (Figs. 1, 2), similar to BM-MSCs [52]. In this study, we confirmed that hAMSCs cultured with PBMC promoted M2 macrophage polarization through the PGE_2_–dependent pathway (Fig. 2).

Subsequently, we successfully developed an efficient strategy for systemic hAMSC treatment using *mdx* mice (Fig. 3). Repeated hAMSC administration resulted in a milder dystrophic muscle phenotype compared to that of untreated mice and exhibited limited mononuclear cell infiltration and muscle interstitium (Fig. 3F), as well as a decreased number of CNFs (Fig. 5C). Notably, the decreased number of CNFs, which represent regenerated fibers following inflammation-induced necrosis, might be caused by the immunosuppressive effect of hAMSCs in DMD muscle. The anti-inflammatory effects of hAMSCs were further confirmed by altered M1/M2 macrophage polarization and cytokine/chemokine expression pattern, e.g., reduced levels of IL-6, mainly secreted by M1 macrophages (Figs. 3, 4). We confirmed that IL-1 receptor antagonist, IL-1ra, in the diaphragm and TIMP-1, which associated with proliferative and anti-apoptotic effects [60], in the TA muscle of hAMSC-treated mice had increased (Fig. 4G, H). Other group reported that partial blocking of IL-1β with IL- 1ra significantly altered only a few behavior-, and strength-related disease parameters in the *mdx* mice, and discussed that treatment with inhibitors that completely block pathways upstream of IL-1β production or combining various inhibitors may produce more favorable outcomes [61]. Therefore, hAMSCs might exert immunomodulatory effects and attenuate the histopathological changes that lead to muscle dysfunction in DMD.

Notably, the long-term benefits of hAMSC treatment were confirmed by the improvement in locomotor activity, grip strength, and cardiac function (Figs. 5, 6). Crucially, hAMSC-treated *mdx* mice ran continuously, implying an improved and sustained motor function. hAMSC-treated *mdx* mice might develop resistance to mechanical stress during running. High concentrations of circulating CK, released from leaked muscle tissue membrane, decreased after hAMSC treatment in *mdx* mice, albeit temporarily (Fig. 3D). Therefore, hAMSCs might protect against physical damage to the muscle. Noteworthy, we demonstrated that hAMSC treatment attenuated DMD progression in young *mdx* mice. On the other hand, the cardiomyopathy in *mdx* mice is mild compared to that occurring in DMD patient [62, 63]. The limiting to provide accurate information to reflect clinical feature in humans, but at least this study suggests a therapeutic effect of hAMSC (Fig. 6). Further investigation using other medium-sized animal model such as CXMD_J_ is expected for studying the cardiac disease phenotype on DMD.

MSC secretions are thought to be the major factors contributing to long-term immunomodulatory effects lasting beyond the short half-life of MSCs. Most growth and other stem cell factors secreted by hAMSCs have tissue healing properties [25]. We demonstrated that mediators secreted by hAMSCs increased with the stimulation in the culture environment by DMD derived myoblasts, such as MCP-1, CXCL-1, G-CSF, IL-6, and IL-8 (Fig. 3A, B). Although MSC secreted mediators contain pro-inflammatory cytokines, seemingly contradicting the immunosuppressive activity of MSCs, such as IL-6 that acts as an anti-inflammatory cytokine as well as an inflammatory cytokine [64]. IL-6 suppresses the secretion of different pro-inflammatory cytokines, including IL-1, TNF-α, GM-CSF, and IFN-γ and induces the production of glucocorticoids, IL- 10, an antagonist of the IL-1 receptor, and a soluble receptor for TNF-α [64]. Previous report showed that growth inhibition of PBMCs in co-culture of BM-MSCs was accompanied by increased COX-2 expression and IDO synthesis in BM-MSCs, through the up-regulation of IL-1, IL-6, IL-8, IFN-γ, MCP-1, and G-CSF with the involvement of direct cell–cell contact between BM-MSCs and PBMCs [65]. In addition, it was also reported that umbilical cord-derived MSCs promote myeloid-derived suppressor cell enrichment by secreting CXCL1 to prevent GVHD after hematopoietic stem cell transplantation [66]. These facts support that the immunosuppression of hAMSCs through the IDO synthesis is similar to that of MSCs in the inflammatory disease. Nevertheless, these features have not been elucidated yet, as studying the long-term benefits of the secretory factors is challenging considering their half-life in cell survival experiments.

We also verified the retention of hAMSCs in the dystrophic muscle after repeated injection, but these were detected for only a short period of time (Fig. 4E, F). Previous study has demonstrated that MSC immunomodulation is host cell-dependent [67]. After infusion, MSCs interact with cytotoxic granules produced by CD8^+^ T and NK cells against recipient MSCs and undergo apoptosis. Apoptotic MSCs are cleared from circulation by the mononuclear phagocyte system. After efferocytosis, phagocytic recipient MSCs produce PGE_2_ and IDO, the final mediators of MSC immunosuppression. Subsequently, MSC apoptosis in the lungs and peripheral immune response impact remote tissue damage [68]. Intravenously administered MSCs undergo apoptosis in the lungs, and apoptotic MSCs and their efferocytosis alter inflammatory pathways in macrophages to exert immunosuppressive effects in severe disease. These facts help us to understand the underlying molecular mechanism of hAMSC therapy, which will likely be elucidated in future studies.

We previously demonstrated the severe inflammatory phenotypes, such as predominant M1 macrophage infiltration with the secretion of pro-inflammatory factors and progressive cardiorespiratory dysfunction, of IL-10 knockout *mdx* mice [36]. Therefore, the immunomodulatory ability of hAMSCs to regulate macrophage activity might prevent the muscle dysfunction caused by muscle fiber inflammation and necrosis. In addition to steroid treatment, several anti-inflammatory treatments ameliorate muscle pathology in a DMD model. For example, TNF-α blockers, such as infliximab [69, 70], and proteasome inhibitors, such as bortezomib, block NF-κB activation following treatment with an adeno-associated virus (AAV) vector encoding short hairpin RNA. Therefore, our hAMSC transplantation strategy could potentially be used in combination with steroids or other immunomodulatory therapies.

We have previously reported a therapeutic strategy for long-term functional recovery in patients with DMD using adult DPSC administration [18]. Furthermore, in this study, we performed a detailed evaluation of motor function and demonstrated the improvement of running endurance for the first time. Other therapeutic approaches to DMD have not revealed such a detailed assessment of motor function, a feature of this paper. For the clinical application of MSC, certain long-term MSC cultivation and expansion risks must be considered; for example, BM-MSCs (passage 3) display a decline in telomerase activity and altered chromosomal morphology with potential anomalous karyotypes, indicating senescence, which results in impaired stem cell stemness and pluripotency [71, 72]. Differences in the immunosuppressive capacity of MSCs between different tissues have been observed because MSC characteristics do not solely depend on species-specific factors but also the tissue source [71]. The characteristics of MSCs isolated from birth-associated tissues depend on their unique biological functions. The immunomodulatory characteristics of hAMSCs isolated from fetal membranes play critical roles in fetal-maternal tolerance. hAMSCs are suitable candidates for the treatment of various diseases, which might be promising for applications in the fields of tissue engineering, regenerative medicine, and cell therapy owing to their unique features, including stem cell properties and low immunogenicity [26, 73]. Additionally, hAMSCs are a new potential cell source for GVHD treatment because they exhibit marked immunosuppression and delay acute GVHD progression by preventing T cell activation and proliferation via the PD-1 pathway, as demonstrated by a recent study [24].

This study is the first to report the therapeutic benefits of hAMSCs for severe muscular disease. Compared to other approaches to treating DMD, our hAMSC therapy strategy resulted in a long-term improvement in locomotor activity. Although further studies are required to elucidate the molecular mechanisms, this approach might be a safe and effective treatment of DMD. Furthermore, our hAMSC transplantation strategy could be combined with steroids, other immunomodulatory therapies, or gene-based therapies using AAV vectors expressing microdystrophin to overcome progressive DMD pathology. The applicability of hAMSCs has attracted clinical interest in treating other muscle diseases associated with chronic inflammation.

## Conclusions

hAMSCs are potential therapeutic agents, as they exert immunosuppressive effects, including modification of macrophage activation. We demonstrated that systemic hAMSC administration, at an early stage in *mdx* mice, can delay the progressive phenotype, such as pathological inflammation and motor dysfunction. The immunosuppressive properties of hAMSCs associated with M2 macrophage polarization might account for the therapeutic effects. This hAMSC treatment strategy could offer therapeutic benefits for patients with DMD.

## Supplementary Information

**Additional file 1:** Characterization of hAMSC and investigation of long-term therapeutic effects on hAMSC-treated *mdx* mouse.

## Data Availability

The datasets used and/or analyzed during the current study are available from the corresponding author on reasonable request.

## Abbreviations

AAV: Adeno-associated virus
ANOVA: Analysis of variance
BM-MSCs: Bone marrow-derived mesenchymal stromal cells
BW: Body weight
CD: Cluster of differentiation
CFSE: Carboxyfluorescein succinimidyl ester
CK: Creatine kinase
CNFs: Centrally nucleated fibers
CXCR: C-X-C Chemokine receptor
CXCL: C-X-C motif ligand
CXMD_J_: Canine X-linked muscular dystrophy model in Japan
dMyHC: Developmental myosin heavy chain
DAB: 3,3′-Diaminobenzidine
DAPI: 4′,6-Diamidino-2-phenylindole
DMD: Duchenne muscular dystrophy
DPSCs: Dental pulp stem cells
EF: Ejection fraction
ELISA: Enzyme-linked immunosorbent assay
FS: Fractional shortening
GAPDH: Glyceraldehyde 3-phosphate dehydrogenase
G-CSF: Granulocyte colony stimulating factor
GDF-9: Growth differentiation factor-9
GM-CSF: Granulocyte-macrophage colony-stimulating factor
GROα: Growth-regulated protein α
GVHD: Graft-versus-host disease
hAMSC: Human amnion-derived mesenchymal stromal cell
HLA-DR: Human leukocyte antigen-DR isotype
HRP: Horseradish peroxidase
H&E: Hematoxylin and eosin
IDO: Indoleamine 2,3-dioxygenase
IGF-1: Insulin-like growth factor-1
IFN: Interferon
IL: Interleukin
IL-1ra: IL-1 receptor antagonist
ITG: Integrin
LV: Left ventricular
LIF: Leukemia inhibitory factor
LVDd: Left ventricular end-diastolic diameter
LVPWd: Left ventricular posterior wall diameter
MCP-1: Monocyte chemotactic protein-1
MHC: Major histocompatibility complex
MMP2: Matrix metallopeptidase 2
MRC1: Mannose receptor C type 1
MSCs: Mesenchymal stromal cells
N.D.: Not detected
PBMC: Peripheral blood mononuclear cell
PGE_2_: Prostaglandin E2
PHA: Phytohemagglutinin
qPCR: Quantitative polymerase chain reaction
ROI: Region of interest
RPMI: Roswell Park Memorial Institute
RV: Right ventricular
SD: Standard deviation
SSEA: Stage-specific embryonic antigens
TA: Tibialis anterior
TIMP-1: Tissue inhibitor of metalloproteinases-1
TNF-α: Tumor necrosis factor-alpha
TGM2: Transglutaminase 2
VEGF: Vascular endothelial growth factor
WT: Wild type
