# Supplementary material for: Immunomodulatory amnion-derived mesenchymal stromal cells preserve muscle function in a mouse model of Duchenne muscular dystrophy
Source: Stem Cell Res Ther. 2023 Apr 27;14:108. doi: 10.1186/s13287-023-03337-0 (PMC10142496; doi:10.1186/s13287-023-03337-0)
Supplement: Supplementary file 1 — Additional file 1: Characterization of hAMSC and investigation of long-term therapeutic effects on hAMSC-treated mdx mouse. [file 13287_2023_3337_MOESM1_ESM.pdf]

Additional files

Figure S1. Morphology and multipotency of hAMSCs.

(A) Cultured hAMSCs (passage 2) had a fibroblast-like morphology. Osteogenic (B), chondrogenic (C), or adipogenic (D) differentiation of hAMSCs was detected using Alizarin red S, Alcian blue, or Oil red O staining, respectively. Original magnification, × 100. Scale bars, 100 μm.

Figure S2. In vitro characterization of hAMSCs.

(A) hAMSCs expressed cell migration-related genes. The mRNA transcripts of cell migration-related genes were detected using real-time PCR comparing normal human dermal fibroblasts (NHDF) and human bone-marrow MSC (hBM-MSC). Data are expressed as the ratio of target gene and *GAPDH* expression. (B) hAMSCs did not increase the rate of CD86- positive cells in hPBMCs. (B, C) hAMSCs were co-cultured with hPBMCs at a ratio of 1:20. CD86 expression in hPBMCs was determined using flow cytometry after 24 h. A representative experiment (B) and the mean ± SD of three independent experiments (C) are shown.

Figure S3. Body weight of hAMSC-treated DMD mice.

(A) Body weight of 20-week-old WT (n = 7), control *mdx* (n = 14), and hAMSC-treated *mdx* (four treatments, n = 10) mice. (B) Body weight of 50-week-old WT (n = 7), control *mdx* (n = 14), and hAMSC-treated *mdx* (four- or six-time of hAMSC treatments; hAMSC-

mdx-4, n = 8; or hAMSC-*mdx*-6, n = 6) mice. All data are presented as the mean ± SD; statistical differences are expressed relative to WT ($^{**}P < 0.01$) mice; ns, not significant; one-way ANOVA (Tukey's post hoc test).

Figure S4. Cytokine and chemokine expression patterns in the diaphragm and hind limb muscle.

Relative expression of cytokines and chemokines in muscle lysates was quantified using the Proteome Profiler™ Array. The array images of diaphragm (left panels) and tibialis anterior (TA) muscle (right panels). The red squares indicate the areas that have been clipped and described in Figure 4E, F. Table shows mouse cytokine array coordinate.

Figure S5. Reverse transcription PCR analysis of human-specific dystrophin expression.

(A, B) To evaluate dystrophin expression in muscles of four-time hAMSC-treated *mdx* mice, RNA was isolated from the tibialis anterior (TA) (A) and diaphragm (B) of WT, control *mdx* (mouse #1, 2, each), and hAMSC-treated *mdx*-4 (TA muscle of mouse #1−5, or #1−3, 4 or 8 weeks post-injection; diaphragm of mouse #1−5, 8 weeks post-injection) mice and human rhabdomyosarcoma (RD) cells and undifferentiated (-) or differentiated (dif) mouse myoblast cells (C2C12) cultured in 2 % horse serum as a control. Reverse transcription PCR was performed using human dystrophin- or mouse *GAPDH*-specific primers. PCR products were separated and analyzed using agarose gel electrophoresis; the arrows indicate dystrophin bands. M, marker (100bp ladder) ; TA, tibialis anterior;

WT, wild type; GAPDH, glyceraldehyde-3-phosphate dehydrogenase.

Figure S6. Histological analysis of the hind limb muscle of hAMSC-treated DMD mice in long-term experiments.

(A) Sirius-red staining of the tibialis anterior (TA) muscle of 1-year-old WT, control *mdx,* and four-time hAMSC-treated *mdx* (hAMSC-*mdx*-4) mice. Original magnification, × 100. Scale bars, 100 μm. (B) Quantification of fibrosis fibers (% of total area) in the TA muscle tissue of WT (n = 2), control *mdx* (n = 4), and four-time hAMSC-treated *mdx* (hAMSC-*mdx*-4, n = 4) mice. (C) Quantification of the ratio of CD206-positive area in the cross-section (% of F4/80 staining area) of the TA muscle of control *mdx* (n = 3−4) and four-time hAMSC-treated *mdx*-4 (n = 3−4) mice. Data are presented as the mean ± SD; statistical differences; ns, not significant; t-test or one-way ANOVA (Tukey's post hoc test).

Figure S7. Histological analysis of the diaphragm of hAMSC-treated DMD mice in long-term experiments.

(A) Hematoxylin and eosin (H&E; left panels) and Masson's trichrome (right panels) staining of the diaphragm of 1-year-old WT, control *mdx,* and six-time hAMSC-treated *mdx*-6 (hAMSC-*mdx*-6) mice. Left panels, original magnification, × 100. Scale bars, 100 μm; Right panels, original magnification, × 160. Scale bars, 200 μm. (B) Quantification of CNFs (% of total area) in the diaphragm of control *mdx* and six-time hAMSC-treated *mdx* (hAMSC-*mdx*-6) mice; n = 3, each group. (C) Quantification of fibrosis fibers (% of

total area) in the diaphragm of control *mdx* and six-time hAMSC-treated *mdx* (hAMSC-*mdx*-6) mice; n = 3, each group. Data are presented as the mean ± SD; statistical differences; ns, not significant; t-test.

Figure S8. Histological analysis of the heart of hAMSC-treated DMD mice in long-term experiments.

(A) Masson's Trichrome staining of the heart of 1-year-old WT, control *mdx*, and four-time hAMSC-treated *mdx* (hAMSC-*mdx*-4) mice. Original magnification, × 27. Scale bars, 2.5 mm. (B) Horseradish peroxidase (HRP)-diaminobenzidine (DAB) immunostaining for F4/80 and CD206 in the cardiac muscle of 1-year-old WT, control *mdx,* and hAMSC-*mdx* mice-4. Original magnification, × 80. Scale bars, 500 µm. Circles indicate DAB-positive areas. (C) Quantification of fibrosis area of the cross-section (% of total area) of the heart of 1-year-old WT (n = 2), control *mdx,* and hAMSC-*mdx*-4 (n = 5, each) mice. (D, E) Quantification of (D) F4/80- and (E) CD206-positive areas in the cross-section (% of total area) of the cardiac muscle of 1-year-old WT (n = 2), control *mdx,* and hAMSC-*mdx*-4 (n = 5, each) mice.

Figure S9. Long-term maintained locomotor activity in hAMSC-treated DMD mice.

(A) Histogram of daily wheel running speed (m/min). The running speed of 1-year-old WT, control *mdx,* and four-time hAMSC-*mdx* mice (hAMSC-*mdx*-4, n = 3, each) mice was calculated by converting m/5 min to m/min and plotted for 2.0 m/min or grater. Table indicates the daily maximum running speed (m/min) and average running speed (m/min).

(B) Horizontal activity per day (m/day) of 1-year-old WT, control *mdx,* and four- and six-time hAMSC-*mdx* mice (hAMSC-*mdx*-4, -6, n = 3, each). (C, D) Circadian rhythm analysis of (C) wheel running distance (m/min) and (D) horizontal activity (m/min) of 1-year-old WT, control *mdx,* and hAMSC-*mdx*-4 (n = 3, each) mice. Data are presented as the mean ± SD; statistical differences are expressed relative to WT ($^{****}P < 0.0001$) and control *mdx* ($^{\#}P < 0.05$) mice; ns, not significant; one-way ANOVA (Tukey's post hoc test).

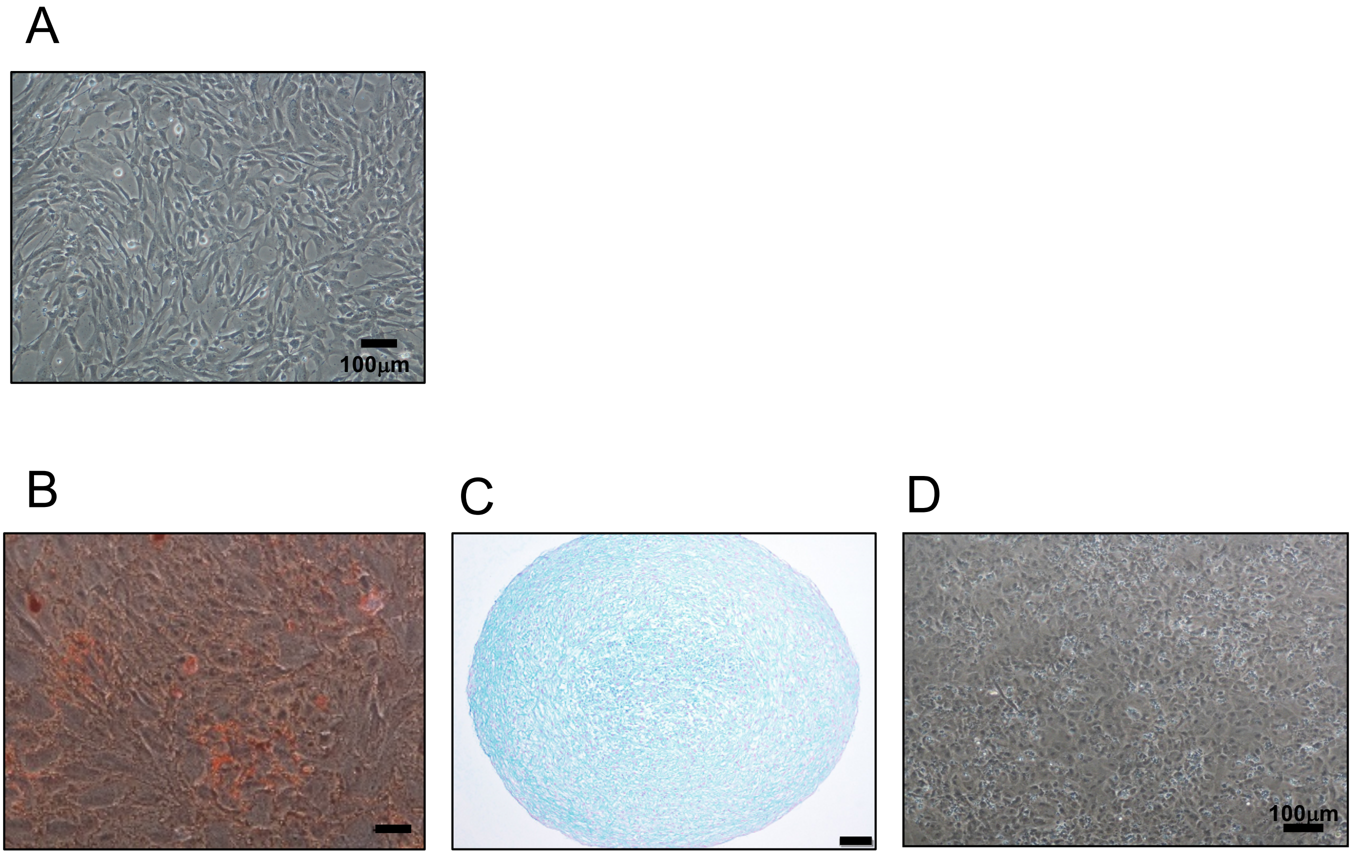

Figure S1

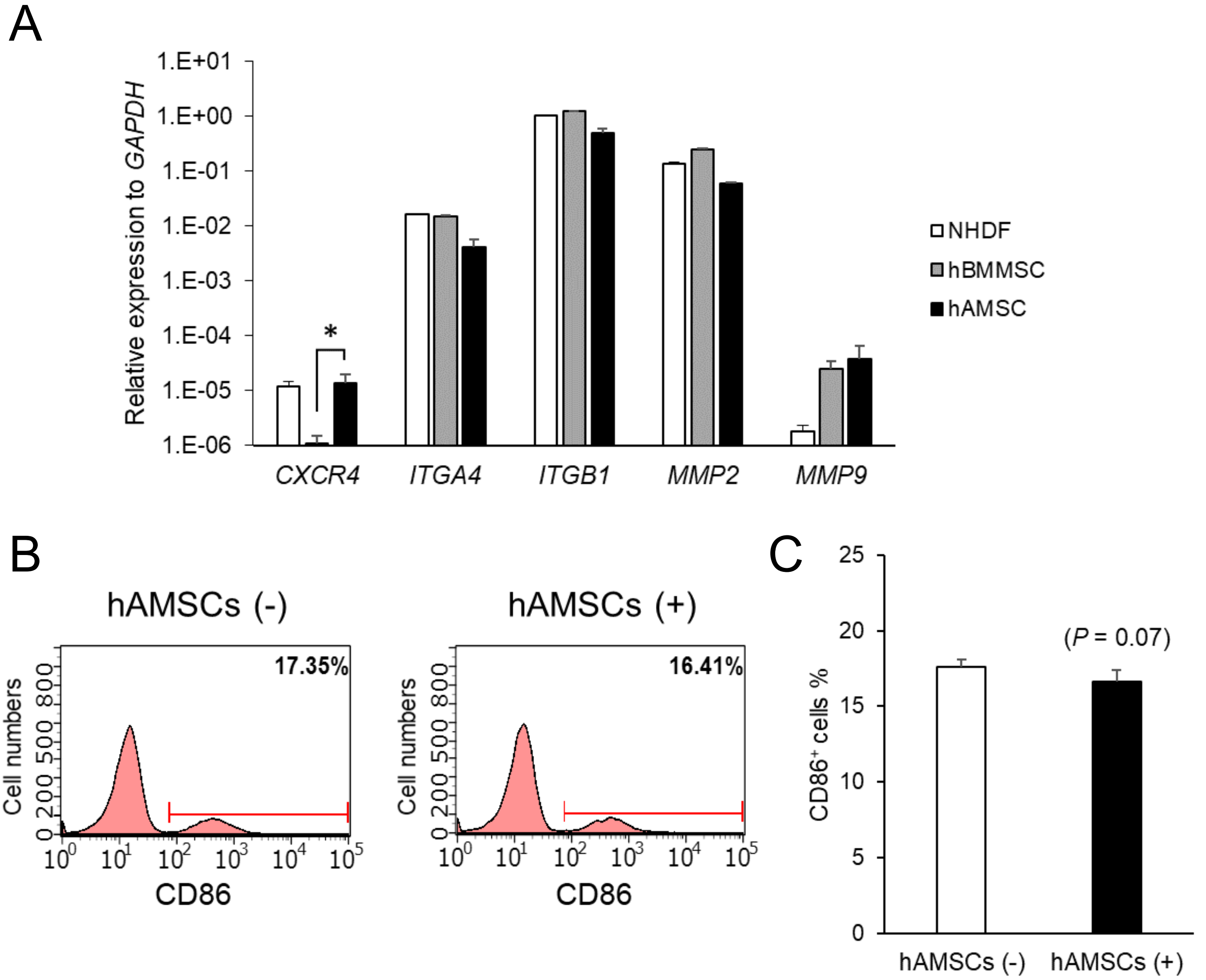
A
1.E+01
1.E+00
1.E-01
1.E-02
1.E-03
1.E-04
1.E-05
1.E-06
Relative expression to GAPDH
*
CXCR4
ITGA4
ITGB1
MMP2
MMP9
NHDF
hBMMSC
hAMSC
B
hAMSCs (-)
hAMSCs (+)
17.35%
16.41%
Cell numbers
CD86
C
25
20
15
10
5
0
CD86+ cells %
(P = 0.07)
hAMSCs (-)
hAMSCs (+)

Figure S2

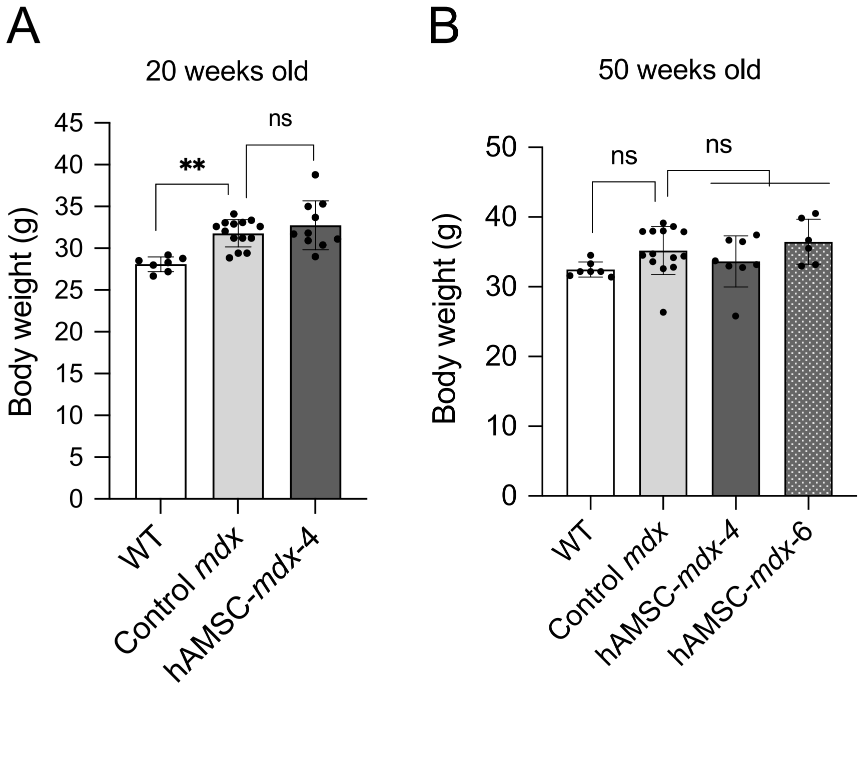
A
20 weeks old
Body weight (g)
45
40
35
30
25
20
15
10
5
0
**
ns
WT
Control mdx
hAMSC-mdx-4
B
50 weeks old
Body weight (g)
50
40
30
20
10
0
ns
ns
WT
Control mdx
hAMSC-mdx-4
hAMSC-mdx-6

Figure S3

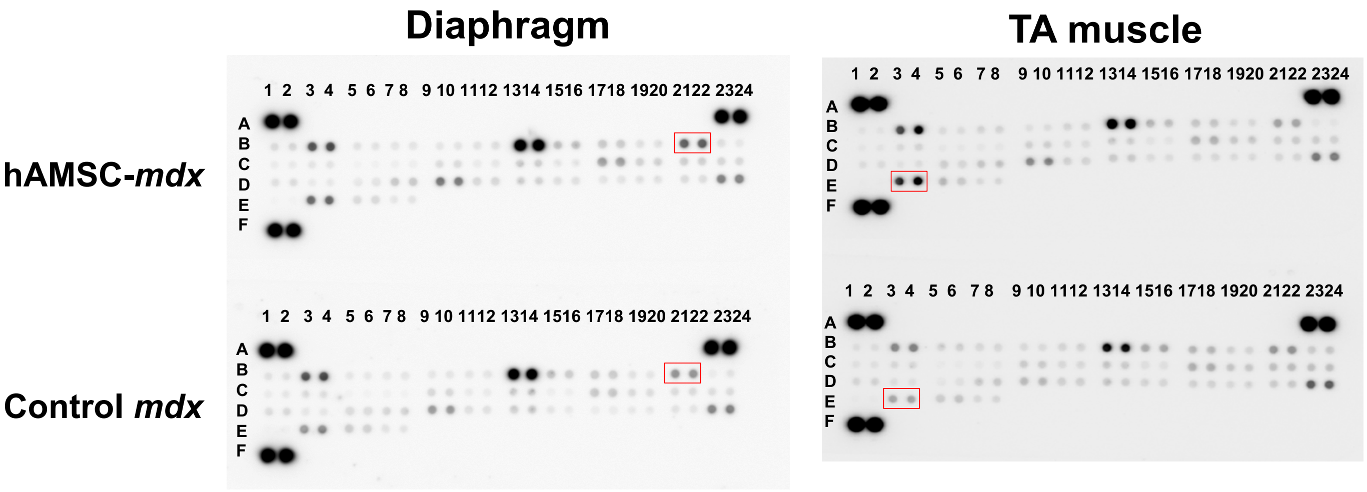

Coordinate	Target/Control	Coordinate	Target/Control	Coordinate	Target/Control
A1,A2	Positive Control	C3, C4	IL-4	D9, D10	JE (CCL2/MCP-1)
A23,A24	Positive Control	C5, C6	IL-5	D11, D12	MCP-5 (CCL12)
B1, B2	BLC (CXCL13/BCA-1)	C7, C8	IL-6	D13, D14	MIG (CXCL9)
B3, B4	C5a	C9, C10	IL-7	D15, D16	MIP-1 (CCL3)
B5, B6	G-CSF	C11, C12	IL-10	D17, D18	MIP-1(CCL4)
B7, B8	GM-CSF	C13, C14	IL-13	D19, D20	MIP-2 (CXCL2)
B9, B10	I-309 (CCL1/TCA-3)	C15, C16	IL-12 (p70)	D21, D22	RANTES (CCL5)
B11, B12	Eotaxin (CCL11)	C17, C18	IL-16	D23, D24	SDF-1 (CXCL12)
B13, B14	sICAM-1(CD54)	C19, C20,	IL-17	E1, E2	TARC (CCL17)
B15, B16	IFN-γ	C21, C22,	IL-23	E3, E4	TIMP-1
B17, B18	IL-1α	C23,C24	IL-27	E5, E6	TNF-α
B19, B20	IL-1β	D1, D2	IP-10 (CXCL10/CRG-2)	E7, E8	TREM-1
B21, B22	IL-1ra	D3, D4	I-TAC (CXCL11)	F1, F2	Positive Control
B23, B24	IL-2	D5, D6	KC (CXCL1)	F23, F24	Negative Control
C1, C2	IL-3	D7, D8	M-CSF		

Figure S4

A

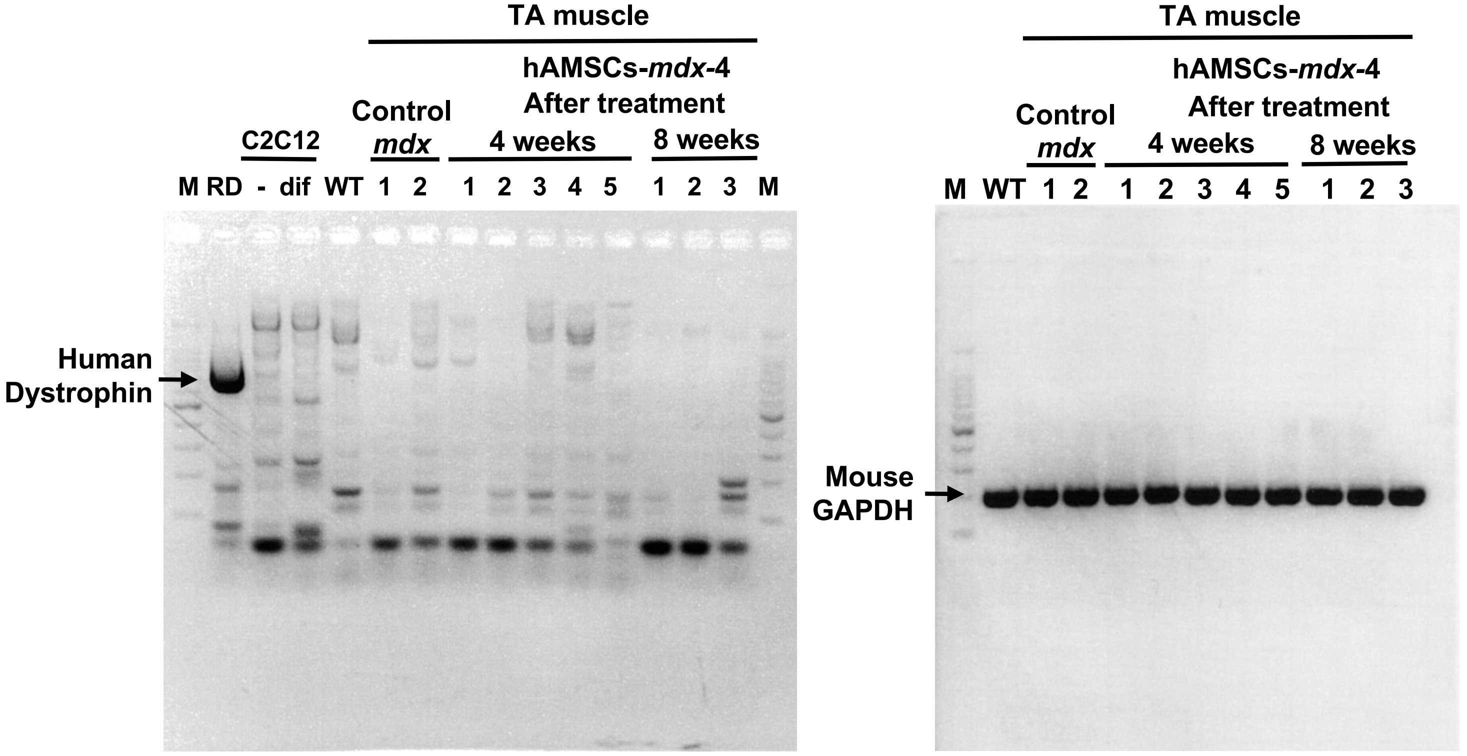
TA muscle
hAMSCs-mdx-4
After treatment
Control
mdx
C2C12
4 weeks
8 weeks
M RD - dif WT 1 2 1 2 3 4 5 1 2 3 M
Human
Dystrophin
TA muscle
hAMSCs-mdx-4
After treatment
Control
mdx
4 weeks
8 weeks
M WT 1 2 1 2 3 4 5 1 2 3
Mouse
GAPDH

B

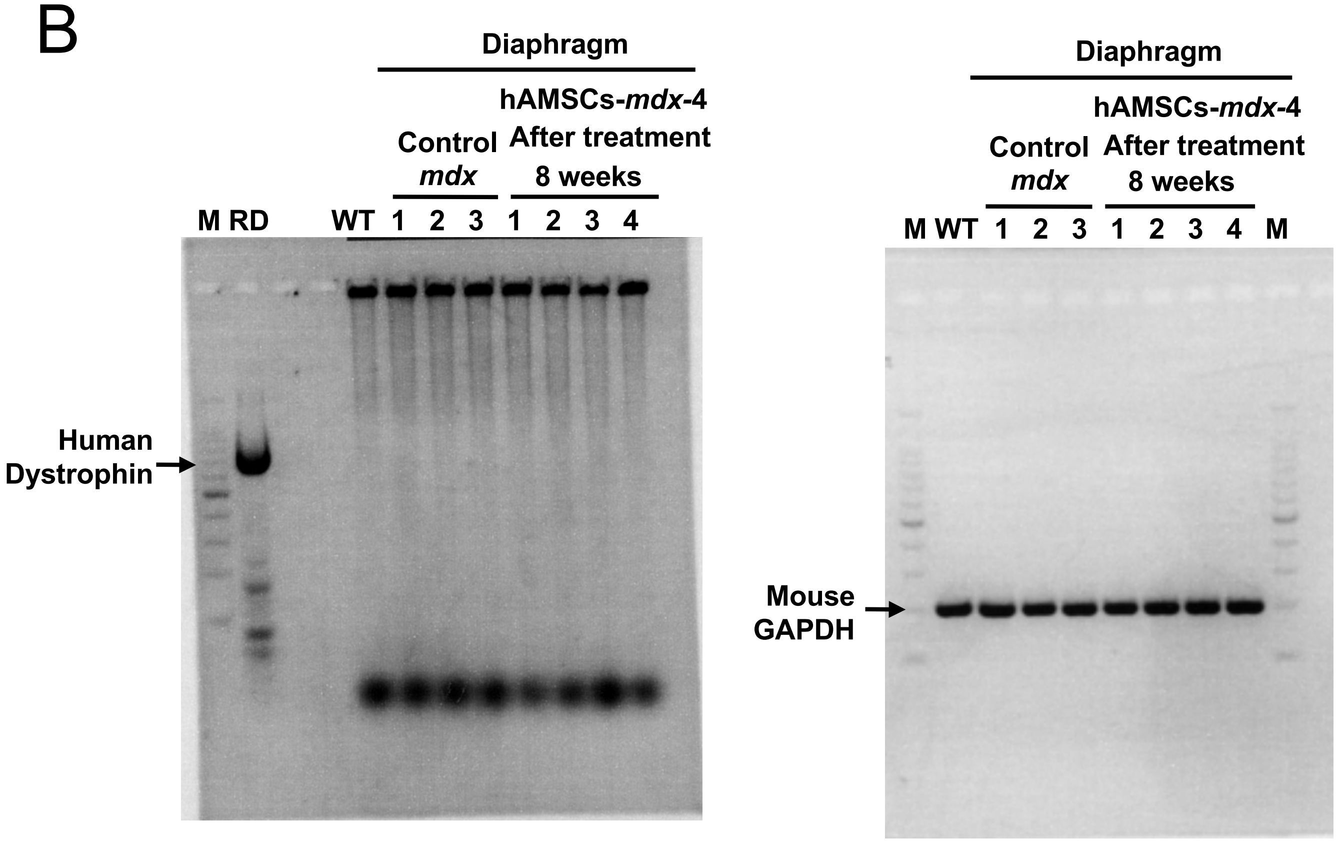
Diaphragm
hAMSCs-mdx-4
Control
mdx
After treatment
8 weeks
M RD WT 1 2 3 1 2 3 4
Human
Dystrophin
Diaphragm
hAMSCs-mdx-4
Control
mdx
After treatment
8 weeks
M WT 1 2 3 1 2 3 4 M
Mouse
GAPDH

Figure S5

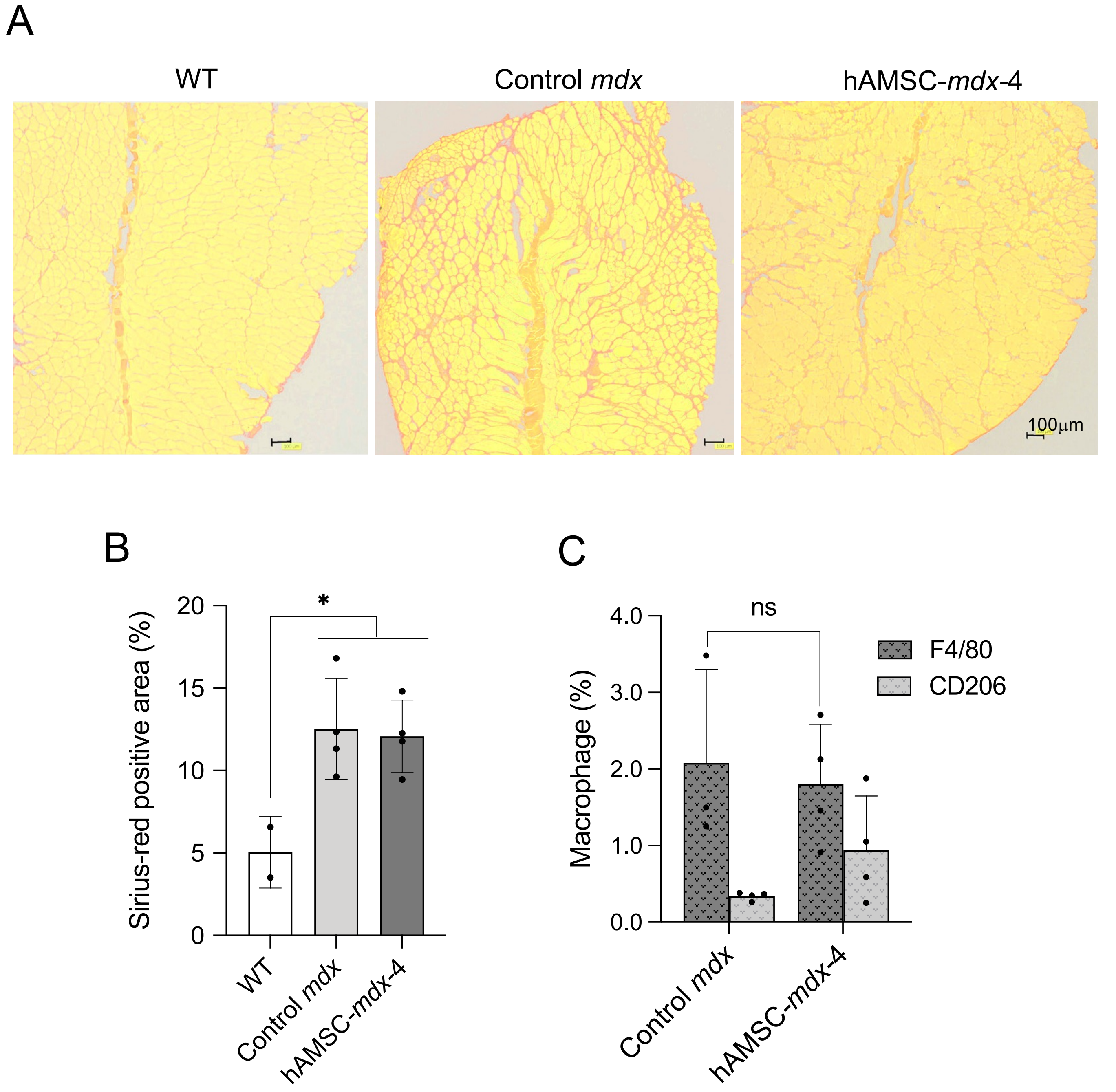
A
WT
Control mdx
hAMSC-mdx-4
100µm
B
Sirius-red positive area (%)
20
15
10
5
0
*
WT
Control mdx
hAMSC-mdx-4
C
Macrophage (%)
4.0
3.0
2.0
1.0
0.0
ns
F4/80
CD206
Control mdx
hAMSC-mdx-4

Figure S6

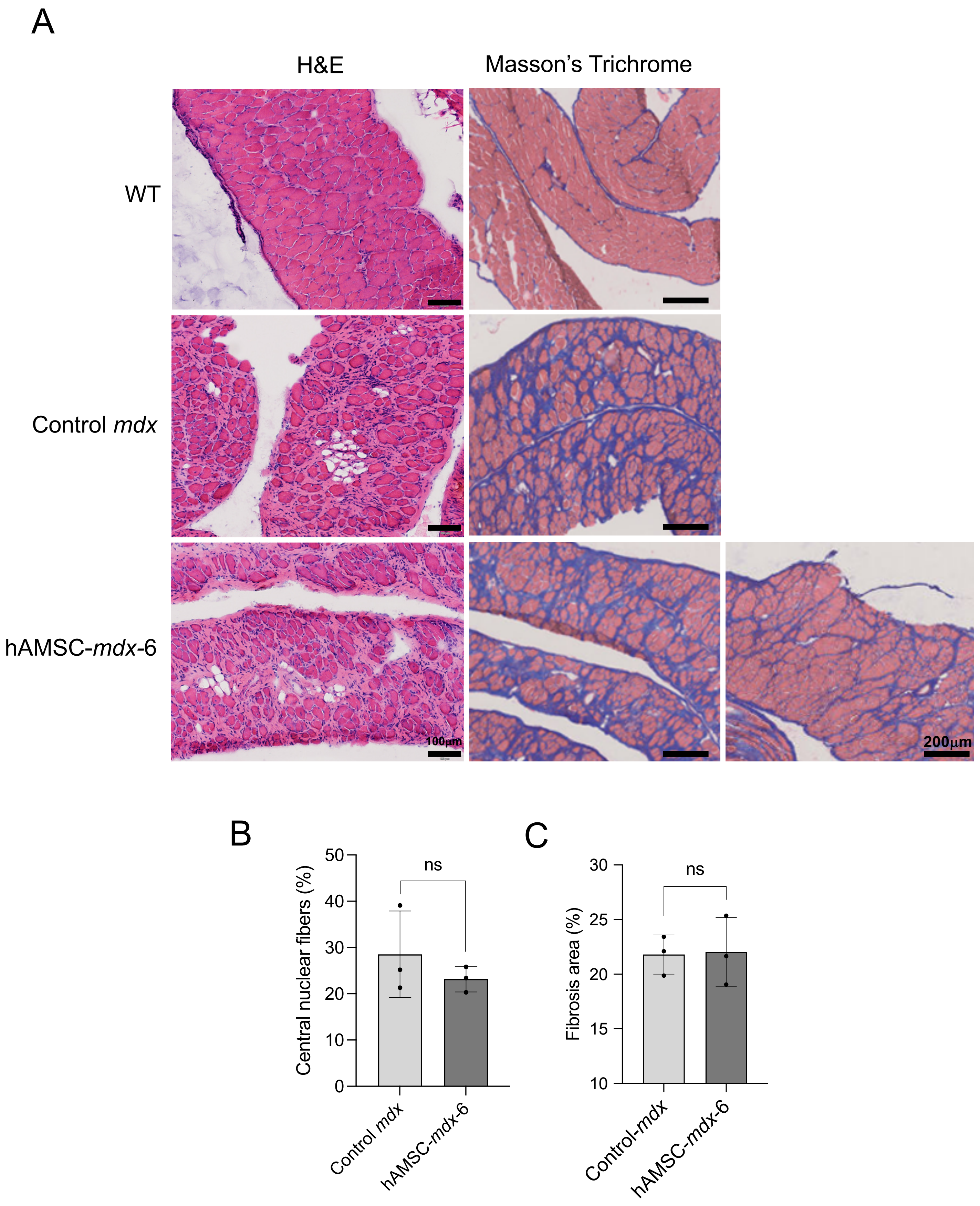
A
H&E
Masson's Trichrome
WT
Control mdx
hAMSC-mdx-6
100µm
200µm
B
Central nuclear fibers (%)
50
40
30
20
10
0
ns
Control mdx
hAMSC-mdx-6
C
Fibrosis area (%)
30
25
20
15
10
ns
Control-mdx
hAMSC-mdx-6

Figure S7

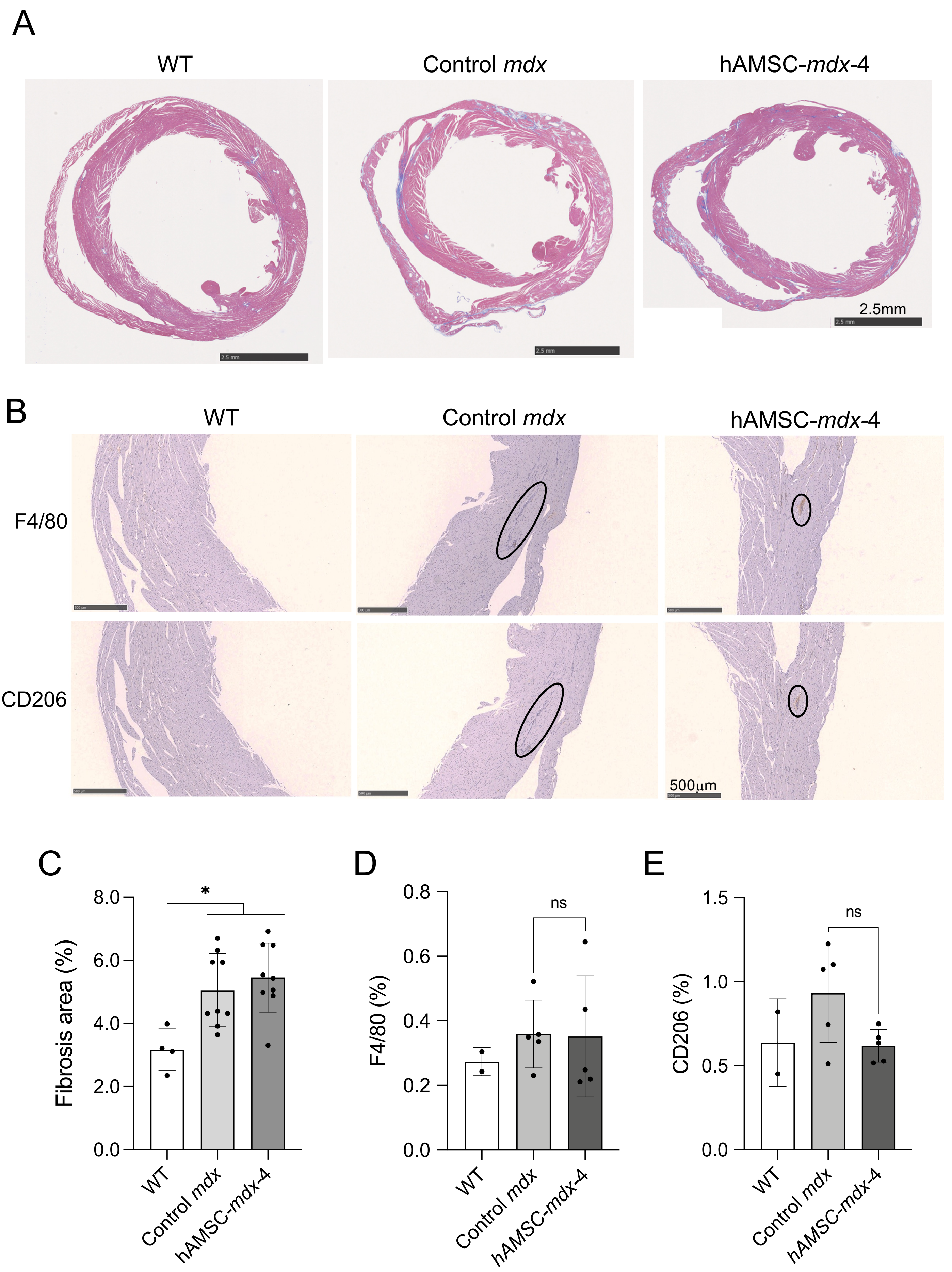
A
WT
Control mdx
hAMSC-mdx-4
2.5mm
B
WT
Control mdx
hAMSC-mdx-4
F4/80
CD206
500µm
C
Fibrosis area (%)
8.0
6.0
4.0
2.0
0.0
*
WT
Control mdx
hAMSC-mdx-4
D
F4/80 (%)
0.8
0.6
0.4
0.2
0.0
ns
WT
Control mdx
hAMSC-mdx-4
E
CD206 (%)
1.5
1.0
0.5
0.0
ns
WT
Control mdx
hAMSC-mdx-4

Figure S8

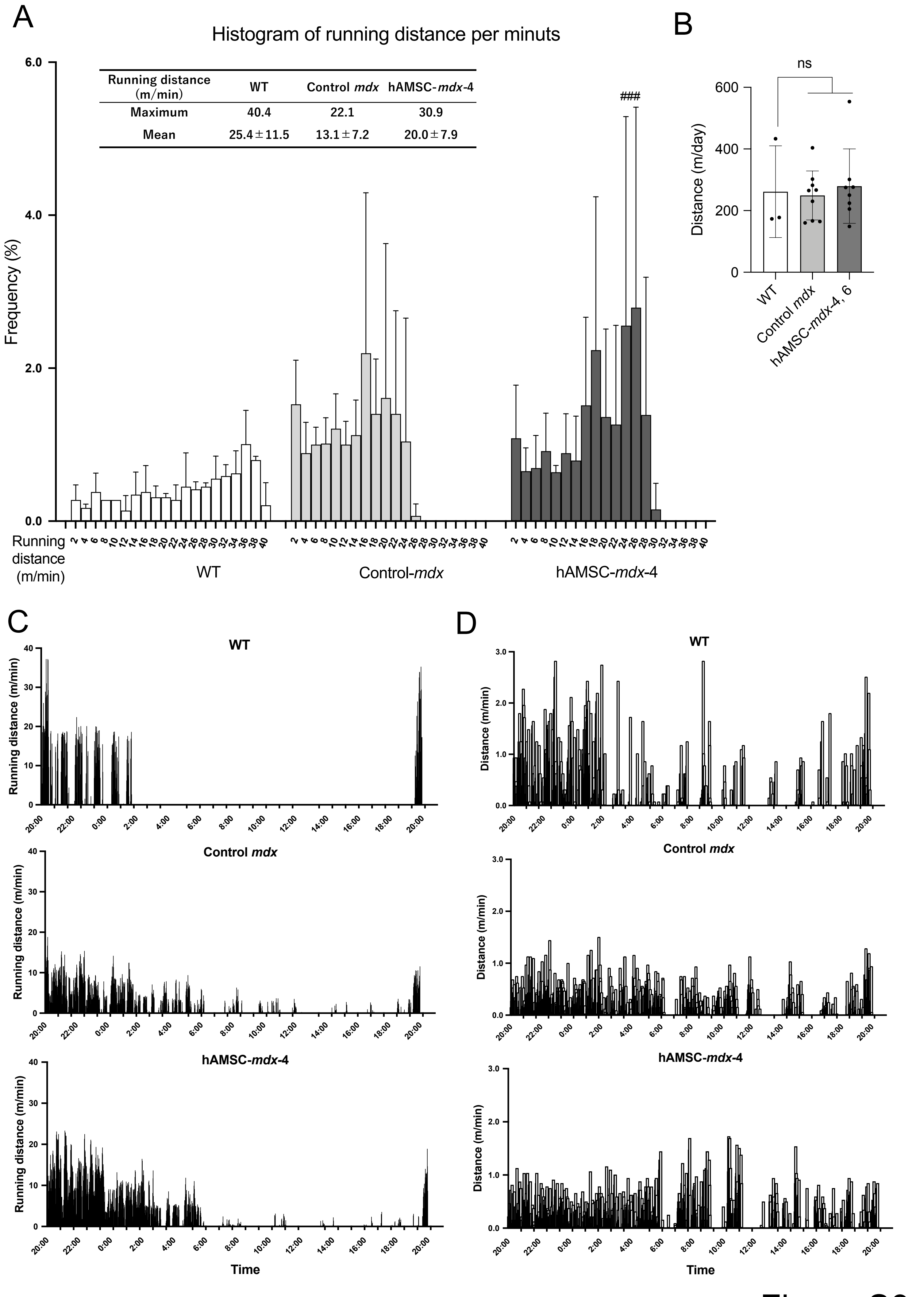
A
Histogram of running distance per minuts
Running distance (m/min) | WT | Control mdx | hAMSC-mdx-4
Maximum | 40.4 | 22.1 | 30.9
Mean | 25.4±11.5 | 13.1±7.2 | 20.0±7.9
###
Frequency (%)
Running distance (m/min)
WT
Control-mdx
hAMSC-mdx-4
B
ns
Distance (m/day)
WT
Control mdx
hAMSC-mdx-4, 6
C
WT
Control mdx
hAMSC-mdx-4
Running distance (m/min)
Time
D
WT
Control mdx
hAMSC-mdx-4
Distance (m/min)
Time

Figure S9
